# Correction: An explainable ensemble approach for advanced brain tumor classification applying Dual-GAN mechanism and feature extraction techniques over highly imbalanced data

**DOI:** 10.1371/journal.pone.0324970

**Published:** 2025-05-20

**Authors:** The PLOS One Staff

## Notice of Republication

This article was republished on May 5, 2025, to correct errors in the formatting of the Figures and Tables that were introduced during the typesetting process. The publisher apologizes for the errors. Please download this article again to view the correct version. The originally published, uncorrected article and the republished, corrected articles are provided here for reference.

## Supporting information

S1 File Originally published, uncorrected article. (PDF)

S2 File Republished, corrected article. (PDF)
