# Supplementary material for: Correction: An explainable ensemble approach for advanced brain tumor classification applying Dual-GAN mechanism and feature extraction techniques over highly imbalanced data
Source: PLoS One. 2025 May 20;20(5):e0324970. doi: 10.1371/journal.pone.0324970 (PMC12091785; doi:10.1371/journal.pone.0324970)
Supplement: S2 File — (PDF) [file pone.0324970.s002.pdf]

PLOS ONE

RESEARCH ARTICLE

# An explainable ensemble approach for advanced brain tumor classification applying Dual-GAN mechanism and feature extraction techniques over highly imbalanced data

**Priyanka Roy**[1,2*], **Fahim Mohammad Sadique Srijon**[1], **Pankaj Bhowmik**[1]

**1** Department of Computer Science and Engineering, Hajee Mohammad Danesh Science and Technology University, Dinajpur, Bangladesh, **2** Department of Computer Science and Engineering, Sylhet International University, Sylhet, Bangladesh

*priyanka03.1802008@std.hstu.ac.bd

OPEN ACCESS

**Citation:** Roy P, Srijon FMS, Bhowmik P (2025) An explainable ensemble approach for advanced brain tumor classification applying Dual-GAN mechanism and feature extraction techniques over highly imbalanced data. PLOS ONE 19(9): e0310748. https://doi.org/10.1371/journal.pone.0310748

**Editor:** Jyotir Moy Chatterjee Graphic Era Deemed to be University, INDIA

**Received:** March 31, 2024

**Accepted:** September 5, 2024

**Published:** September 27, 2024

**Copyright:** © 2024 Roy et al. This is an open access article distributed under the terms of the Creative Commons Attribution License, which permits unrestricted use, distribution, and reproduction in any medium, provided the original author and source are credited.

**Data Availability Statement:** The data underlying the results presented in the study are available from (https://www.kaggle.com/datasets/masoudnickparvar/brain-tumor-mri-dataset and https://www.kaggle.com/datasets/niranjananehru/brain-tumour-dataset).

**Funding:** The author(s) received no specific funding for this work.

## Abstract

Brain tumors are one of the leading diseases imposing a huge morbidity rate across the world every year. Classifying brain tumors accurately plays a crucial role in clinical diagnosis and improves the overall healthcare process. ML techniques have shown promise in accurately classifying brain tumors based on medical imaging data such as MRI scans. These techniques aid in detecting and planning treatment early, improving patient outcomes. However, medical image datasets are frequently affected by a significant class imbalance, especially when benign tumors outnumber malignant tumors in number. This study presents an explainable ensemble-based pipeline for brain tumor classification that integrates a Dual-GAN mechanism with feature extraction techniques, specifically designed for highly imbalanced data. This Dual-GAN mechanism facilitates the generation of synthetic minority class samples, addressing the class imbalance issue without compromising the original quality of the data. Additionally, the integration of different feature extraction methods facilitates capturing precise and informative features. This study proposes a novel deep ensemble feature extraction (DeepEFE) framework that surpasses other benchmark ML and deep learning models with an accuracy of 98.15%. This study focuses on achieving high classification accuracy while prioritizing stable performance. By incorporating Grad-CAM, it enhances the transparency and interpretability of the overall classification process. This research identifies the most relevant and contributing parts of the input images toward accurate outcomes enhancing the reliability of the proposed pipeline. The significantly improved Precision, Sensitivity and F1-Score demonstrate the effectiveness of the proposed mechanism in handling class imbalance and improving the overall accuracy. Furthermore, the integration of explainability enhances the transparency of the classification process to establish a reliable model for brain tumor classification, encouraging their adoption in clinical practice promoting trust in decision-making processes.

**Competing interests:** The authors have declared that no competing interests exist.

## Introduction

The human brain, a complicated organ, serves as the epicenter of the central nervous system, directing an array of cognitive, sensory, and motor functions [1]. It is a vital organ and is also vulnerable to many diseases including brain tumors. A brain tumor is an abnormal growth of cells within the brain or the central spinal canal [2]. These tumors can be benign (non-cancerous) or malignant (cancerous) and can originate from brain tissues or result from cancer elsewhere in the body that has spread to the brain. Brain tumors make up approximately 85% to 90% of all initial central nervous system (CNS) tumors [3]. Worldwide, they affect a significant number of people, with hundreds of thousands of new cases reported each year. In 2023, it has reached even higher bars and the rate has increased by 1.8% [4]. More than any other type of cancer, brain tumors have long-term, life-altering effects on a patient's physical, mental, and emotional health. The mortality rate associated with certain types of brain tumors is alarmingly high. Within the United States exclusively, the collective mortality attributed to cancer is approximated at 24,810 individuals, disaggregated into 14,280 male fatalities and 10,530 female fatalities [5]. Therefore, it is very essential to address the need for precise detection and effective treatment.

In recent years, many studies have been done in the medical domain by using the advancement of artificial intelligence (AI). Different machine learning (ML) techniques including deep learning have been used to classify brain tumors. Brain tumor classification with machine learning on Magnetic Resonance Imaging (MRI) or Computerized Tomography (CT) images faces several challenges. Among these, a major obstacle is the highly imbalanced nature of brain tumor datasets. The limited access and availability to the medical data leads to insufficient data and hence, imbalanced class distribution. This leads to inaccurate model prediction due to the biased behavior of the classifier towards the majority class. While a few previous research have contributed to addressing data imbalance mostly for binary classification tasks, our study focuses on the challenging scenario of highly imbalanced datasets used in classifying multiple classes of brain tumors. Moreover, the accuracy that has been achieved to date reflects that a more reliable and stable model is needed. We aim to contribute by investigating advanced techniques that utilize the power of deep learning and feature extraction methods to enhance brain tumor classification, thereby paving the way for improved early detection and intervention. There has been very little work on classifying brain tumor disease in any case with imbalanced datasets. Our study is motivated by the scope for investigation in this scenario to ensure that brain tumor classification can be conducted without model bias even with the possibility of class imbalance in a multiclass dataset. To mitigate the existing shortcomings, our research intends to make the following contributions:

1. Propose a novel Deep Ensemble Feature Extraction (DeepEFE) model to enhance the early detection and diagnosis of brain tumors.
2. Propose a hybrid dual-GAN mechanism combining Real-ESRGAN and WGAN-GP to mitigate data imbalance issues and enhance the training process of the deep neural networks (DNN).
3. Establish a generalized pipeline that provides better performance compared to other existing models in terms of Accuracy, Precision, Sensitivity, F1-Score, and other performance standards by utilizing advanced deep learning techniques.
4. Facilitate model explainability through the eXplainable Artificial Intelligence (XAI) technique to ensure the transparency of the proposed model.
5. Contribute to the advancement of medical knowledge and healthcare standards in the field of brain tumor classification, ultimately leading to better patient outcomes and more reliable diagnostic procedures.

The remainder of the paper is structured as follows: Background and Related Works examines relevant works in this field highlighting the need for future investigation, Methodology outlines the ultimate proposed pipeline to effectively handle these limitations, and Experimental Results and Discussions presents the detailed findings along with further analyses and discussions. Finally, the Conclusion and Future Work section wraps up the work and provides future recommendations.

## Background and related works

There have been many research works in the domain of brain tumor classification. The adaptive pillar K-means algorithm is used for segmentation purposes and a two-tier classification approach is used for the classification of brain tumors in one study using a dataset that is available online [6]. The highest accuracy the study has achieved is 96.6%. Afshar et al. proposed a deep learning approach titled "BoostCaps: A Boosted Capsule Network for Brain Tumor Classification" [7]. They have used a brain tumor dataset from Figshare consisting of 3064 T1-weighted contrast-enhanced images [8]. The proposed BoostCaps network has surpassed other single capsule networks with an accuracy of 92.45%. Shahzadi et al. discussed the classification of brain tumors, specifically gliomas, into High Grade (HG) and Low Grade (LG) [9]. It introduces a method that combines Convolutional Neural Networks (CNNs) and Long Short Term Memory (LSTM) networks to classify 3D brain tumor MR images. The authors used the pre-trained VGG16 model for feature extraction on the BraTS 2015 dataset, which outperformed other feature extraction models (ResNet and AlexNet). They achieved an 84% accuracy in glioma classification [10].

Meanwhile, a study investigated the application of wrapper-based metaheuristic deep learning networks (WBM-DLNets) feature optimization algorithms [11]. The study validated the proposed WBM-DLNets approach using an online dataset, with results demonstrating significant improvements in classification accuracy compared to using the full set of deep features [12]. The dataset has 826, 822, 395, and 827 images of Glioma, Meningioma, No Tumor, and Pituitary Tumor classes respectively. The DenseNet-201-GWOA and EfficientNet-b0-ASOA combinations achieved the highest accuracy of 95.7%. Sujatha & Rao explored the automation of brain tumor type detection from MRI images using a customized DenseNet201 deep neural network [13]. They worked with a dataset comprising 7023 brain tumor MRI images across four classes ('no tumor,' 'glioma,' 'meningioma', and 'pituitary') and fine-tuned the model by adapting the final layer to predict these classes. The model achieved 88% accuracy during testing. Bitto et el. proposed a Tumor-Net to classify brain images where they explored several deep learning algorithms (VGG16, VGG-19, ResNet50, Xeption, InceptionNet-V3) [14]. Although the ResNet-50 achieved the highest classification accuracy (96.67%), the study was conducted on a balanced dataset consisting of only three classes.

Pilaoon et al. employed deep learning convolutional neural networks (CNN) for the binary classification of GBM brain tumors [15]. The dataset consisted of 155 MRI images from the REMBRANDT database. Transfer learning, utilizing pre-trained networks such as GoogleNet and AlexNet, was employed to distinguish GBM brain tumors from normal brain tissue. The results showed that GoogleNet achieved an 80.85% accuracy, while AlexNet achieved a higher accuracy of 93.62%. Najeeb et al.'s work emphasized the limitations of manual identification [16]. The study used deep learning networks and a Kaggle dataset for brain tumor segmentation. Transfer learning, U-Net, and Resnet architectures were used for multi-classification. The results demonstrated that this research framework outperformed existing methods, with Resnet achieving the highest accuracy of 90.2% in brain tumor classification. Toğaçar et al. introduced BrainMRNet, a convolutional neural network model using attention modules and hypercolumn technique for brain tumor detection from MRI images [17]. BrainMRNet

excelled compared to pre-trained models like AlexNet, GoogleNet, and VGG16, achieving a classification success of 96.05%. Shree & Kumar worked towards the enhancement of brain tumor detection accuracy in MRI images through a multi-step approach [18]. It involved image preprocessing, morphological operations, and feature extraction methods like Discrete Wavelet Transform (DWT) and Gray-Level Co-occurrence Matrix (GLCM) features. A Probabilistic Neural Network (PNN) classifier was employed to distinguish between normal and abnormal brain tissues. The approach achieved 95% accuracy in identifying abnormal brain tissues. A hybrid feature extraction method with a regularized extreme learning machine (RELM) was used in a study for precise brain tumor classification [19]. The approach involved feature extraction from brain images from the Figshare dataset using a hybrid method, followed by the computation of the covariance matrix and projection using principal component analysis (PCA). Finally, the RELM classifier is employed for tumor classification which achieved an average accuracy of 92.61%.

VGG19 combined with Inverted Pyramid Pooling Module (iPPM) were utilized in a research investigation to classify brain tumors [20]. After intensive parameter optimization and 3-fold cross-validation, Adam optimizer provided the best classification accuracy of 99.3% on a balanced dataset with four types of tumor diseases' images. Another study explored the classification of epileptic seizure in the brain by conducting deep-learning models on electroencephalogram (EEG) data [21]. Applying CNN's feature extraction power and LSTM's classification capacity, the study obtained an accuracy of 99.48%. Adaptive histogram contrast normalization with learning-based neural quantization (AHCN-LNQ) was used in a study where the AHCN was used as a preprocessing and noise-removing technique [22]. Image segmentation by Otsu threshold was conducted for feature extraction purposes. The study's proposed model achieved an accuracy of 93%. The issue of data imbalance wasn't reflected in the paper, however.

Brain tumor classification using gene expression has also been studied. Classified gene expression data that has two classes of brain tumors was investigated in a research work [23]. The study incorporated an optimization technique named PSCS. The metaheuristic algorithm such as the Cuckoo Search (CS) algorithm's effectiveness in improving the classification task was also explored in this study. This study's proposed methodology achieved an accuracy of 98.7% in classifying between the two tumor types. However, the study faced several constraints including hyperparameter sensitivity, lack of generalizability, complexity in interpretability due to the nature of gene-based data, and associated time cost for identifying the optimal hyperparameter set. Since redundant and unnecessary genes can pose a hindrance to accurately classifying the disease, it is necessary to identify the meaningful features of the genes. Such an initiative with a feature selection technique has been adopted in research work to classify breast cancer disease [24]. To generate optimal features and increase outcome accuracy, the Sine Cosine Algorithm (SCA) was combined with the CS algorithm. Utilizing the privileges of SCA's local search and CS's global search functionality, their proposed model achieved an accuracy of 99.62%. However, the study did not address the cost management of the feature optimization process. Additionally, in the compared algorithms section, there is a writing anomaly about the fitness function definition.

Instead of SCA, the Spider Monkey Optimization (SMO) was combined with CS by a group of researchers [25]. This study utilized an additional gene feature-reducing process named minimum redundancy maximum relevance (mRMR). It used 8 high-dimensional datasets consisting of gene information for several cancer diseases and achieved an average mean classification accuracy of 98.68% without considering the deviations. Like the previous studies on gene-feature selection, it was prone to parameter sensitivity. It was a resource-intensive model.

Another investigation was conducted on different combinations of CS and SMO [26]. One method (CSSMO) was utilizing CS followed by SMO. Another (SMOCS) was the reverse of the previous one, SMO followed by CS. To reduce gene expression redundancy, mRMR was also leveraged in this study. Six different cancer datasets were evaluated with the proposed models and 96.52% was the highest accuracy which was achieved in classifying Leukemia by the second method (SMOCS). All these studies on gene expression data faced some common limitations that are high dimensionality in datasets, redundant features, and computational cost. Such analysis with genetic data might not cover the scenario of all adjacent cells and thus could lose precision in classification [27].

Optimal feature selection through different metaheuristic algorithms was investigated for the detection of COVID-19 disease along with a newly proposed algorithm named Marine Predator Chaotic Algorithm (MPCA) [28]. 96.27% mean accuracy was achieved with the proposed MPCA algorithm at the cost of computation cost.

Many researchers focused on using the generative adversarial network framework (GAN). A Generative Adversarial Network (GAN) is a deep learning model comprising a generator and discriminator that engage in a competitive process to create and distinguish realistic data, primarily used for image generation and enhancement tasks [29]. Research has been conducted on the Figshare dataset using a progressive-growing generative adversarial network (PGGAN) [30]. By augmenting brain tumor image data, employing a soft voting approach, and using balancing techniques, this approach compared different GAN techniques. The research highlighted PGGAN as the most suitable GAN technique for data generation. Han et al. also conducted extensive work using PGGAN [31]. Their proposed approach consists of a two-step GAN-based Data Augmentation (DA) method: PGGANs and Multimodal UNsupervised Image-to-image Translation (MUNIT). The study explored various combinations of these GAN-based DA approaches and classic data augmentation. The combination of GAN-based DA with classic DA outperformed other approaches.

Limited training data and incomplete MRI collections from various modalities can impact deep learning performance. Ge et al. proposed a novel pairwise Generative Adversarial Network (GAN) architecture to generate synthetic brain MRIs for missing modalities, aiming to enhance the training dataset and mitigate overfitting [32]. The main objectives included the introduction of a cross-modality GAN-based image generation method, a two-stage training strategy combining GAN-augmented and real MRIs for glioma classification, and experimental validation on an open TCGA dataset, resulting in a 2.57% improvement in glioma classification accuracy [33]. This dataset contains segmentation labels for pre-operative Low-Grade Glioma (LGG) scans from The Cancer Genome Atlas. These scans include various MRI modalities and have been processed to extract radiomic features. While the synthetic images slightly differed statistically from real ones, the proposed method proved effective in offering enhanced deep learning performance in glioma classification. A study introduced an ensemble approach for brain tumor classification employing a range of pre-trained CNNs to extract the deep features from brain MR images, which were then evaluated by various machine learning classifiers [34]. Three datasets were used: the first two were from the Kaggle website, and the other one was the previously mentioned Figshare dataset [35, 36]. Notable findings included the suitability of DenseNet-169 for small datasets (accuracy of 98.04%) and the effectiveness (accuracy of 98.83%) of an ensemble of DenseNet-121, ResNeXt-101, and MnasNet for the second dataset.

Several limitations were observed in the reviewed work as some are depicted in Table 1. Some models demonstrated lower testing phase accuracy, indicating the need for improved model performance. Limited sample sizes in the datasets could potentially restrict the model's generalizability. High model complexity was seen in some studies, which could impact their practical applicability. Most of the studies used fewer classes. No study focused on how to address the

**Table 1. Some recent benchmark studies.**

| Article | Year | Dataset | Model | Accuracy | Limitation(s) |
|---|---|---|---|---|---|
| 7 | 2020 | Balanced (3 classes) | BoostCaps | 92.45% | Validation missing and not prepared for imbalanced datasets |
| 13 | 2023 | Balanced (4 classes) | DenseNet201 | 88.00% | Data-imbalance issue not addressed and poor performance |
| 14 | 2023 | Balanced (4 classes) | ResNet-50 | 96.76% | Further scope for enhancing performance and and no focus on how to deal with real-life imbalanced datasets |
| 23 | 2023 | Balanced Dataset (2 classes) | Particle Swarm Cuckoo Search (PSCS) | 98.70% | Hyperparameter sensitivity, lack of generalizability, and complexity in interpre- tability |
| 24 | 2023 | Gene Expression Data (2 classes) | Sine Cosine Algorithm (SCA) | 99.62% | Cost intensive, high dimensional data, feature redundancy |
| 25 | 2023 | Gene Expression Data (2 classes) | Spider Monkey Optimization (SMO) and Cuckoo Search (CS) | 98.68% | Prone to parameter sensitivity and a resource intensive model |
| 26 | 2024 | Gene Expression Data (2 classes) | SMO followed by CS | 96.52% | Cost intensive, high dimensional data, lack of generalizability, sensitivity to model parameter tuning, and time-consuming model |
| 28 | 2024 | Numerical Datasets | Marine Predator Chaotic Algorithm (MPCA) | 96.27% | Cost intensive |
| 30 | 2023 | Balanced (3 classes) | PG-GAN | 98.85% | Did not address data imbalance issues and the proposed model was not stress-tested. |
| 34 | 2021 | 3 Balanced datasets (2, 2, and 4 classes) | Ensemble of Deep Features with SVM | 98.83% | No generalized pipeline or model proposed for brain tumor prediction as different models performed well for different datasets with different sizes. Moreover, lacks explaining why the FC CNN model performed better while extracting features |

The table provides insights into some of the state-of-the-art research works and the research gaps found by the current investigation. Notably, one common limitation is that no research addressed the issue of data imbalance. Additionally, no previous study focused on establishing a transparent generalized pipeline for brain tumor classification by incorporating advanced GAN techniques and XAI.

https://doi.org/10.1371/journal.pone.0310748.t001

imbalanced dataset. Failure to address data imbalance issues may lead to biased results and hinder the classification process. Explanability to the model's performance wasn't found in any of the studies when reviewing the studies. Considering these shortcomings and understanding the need for more reliability this research comes with the following propositions:

1. The data imbalance issue, particularly between the majority class and the minority class within the MRI image datasets, needs to be addressed to create a more generalized model. To handle the data imbalance issue, different methods can be used such as the Generative Adversarial Networks (GANs).
2. Optimizing the quality and relevance of the dataset should be conducted appropriately by exploring and implementing advanced preprocessing methods that aim to improve data quality, reduce noise, and enhance the model's capacity to extract meaningful features from the input images.
3. Working with more diverse datasets should be ensured to secure model reliability and explore a broader scope.
4. Utilizing different efficient models such as the deep feature extraction methods should be explored to introduce a generalized and efficient pipeline for brain tumor classification that can aid the development of medical diagnostic approaches.

## Methodology

The methodology section of our research paper provides a broad explanation of the research process, describing the distinct stages and methodologies employed for the purpose of brain tumor classification.

## Overview of the proposed methodology

Fig 1 illustrates the comprehensive methodology proposed by this study. This provides a detailed insight into the used components and steps taken. This study applies basic image preprocessing and augmentation techniques to the target image dataset, enhancing its quality and usability for further analysis and interpretation. We introduce a hybrid framework that combines Wasserstein GAN with Gradient Penalty (WGAN-GP) and Real Enhanced Super-Resolution GAN (Real-ESRGAN) to effectively address the issue of data imbalance by generating real-like artificial data images. Addressing data imbalance is crucial prior to the

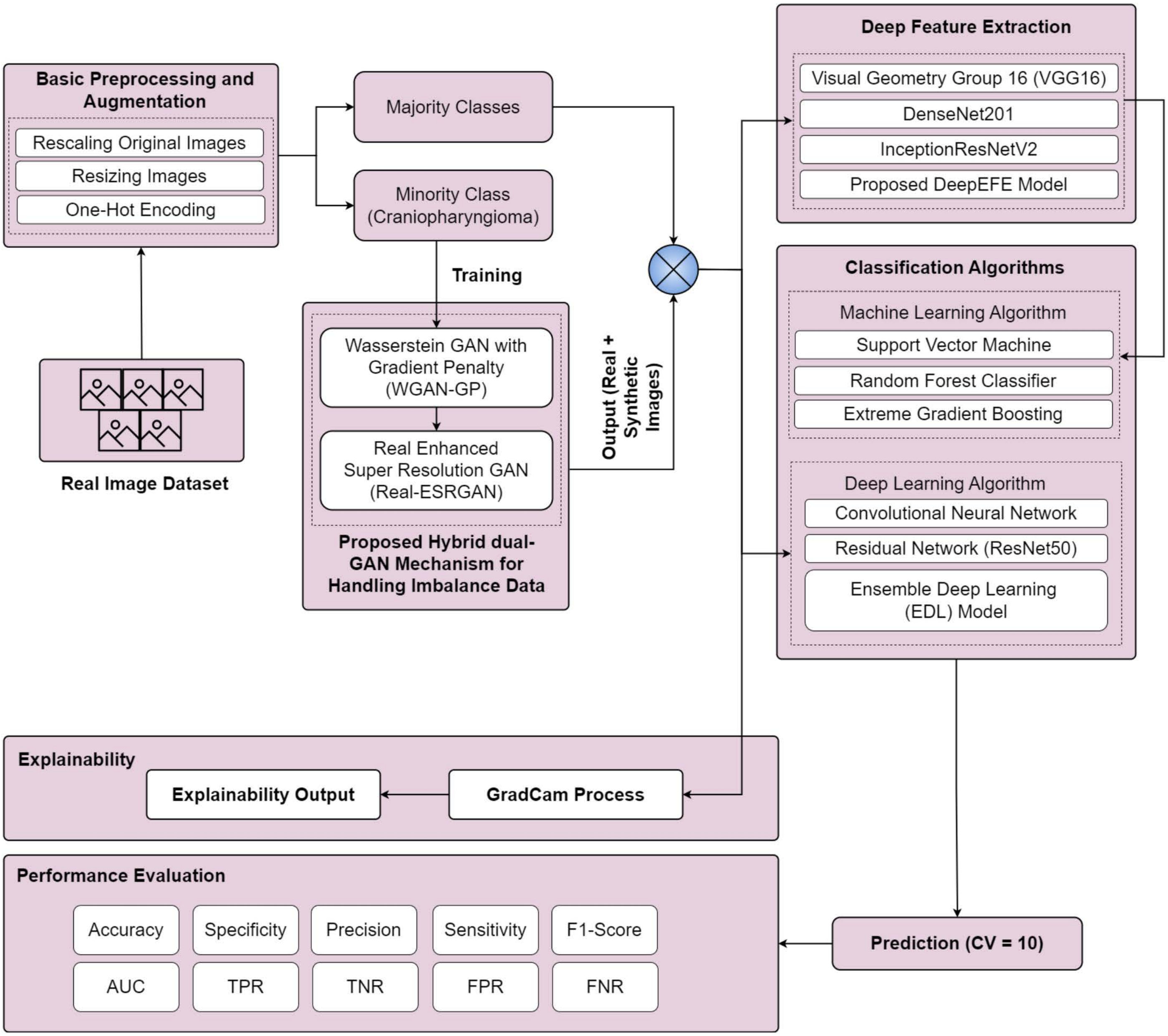


**Fig 1. Proposed methodology.**

https://doi.org/10.1371/journal.pone.0310748.g001

application of classification algorithms, as it serves to reduce the risk of model bias and unfavorable outcomes.

To ensure quality performance in early-stage brain tumor detection, this study incorporates an advanced deep feature extraction technique. Visual Geometry Group 16 (VGG16), DenseNet201, and InceptionResNetV2 models, referred as the base models, are blended together to implement a novel Deep Ensemble Feature Extraction (DeepEFE) model framework. Traditional ML classifiers such as Support Vector Machine (SVM), Random Forest (RF), and eXtreme Gradient Boosting (XGB) classifiers are applied to make the ultimate prediction at the output layer after the intensive feature extraction phase. In addition to investigating various feature extraction models, this research delves into state-of-the-art deep learning models such as Convolutional Neural Network (CNN), Residual Network with 50 layers (ResNet50), and an Ensemble Deep Learning (EDL) model to thoroughly analyze the prediction outcomes. Furthermore, the robustness and generalizability of the proposed pipeline is established through an intensive 10-fold cross validation. Ultimately, this research focuses on exploring Grad-CAM as an explainable AI tool to further demonstrate the outcomes by turning opaque algorithms into understandable models. The detailed flow of the adopted methodology can be viewed from the Fig 2 below.

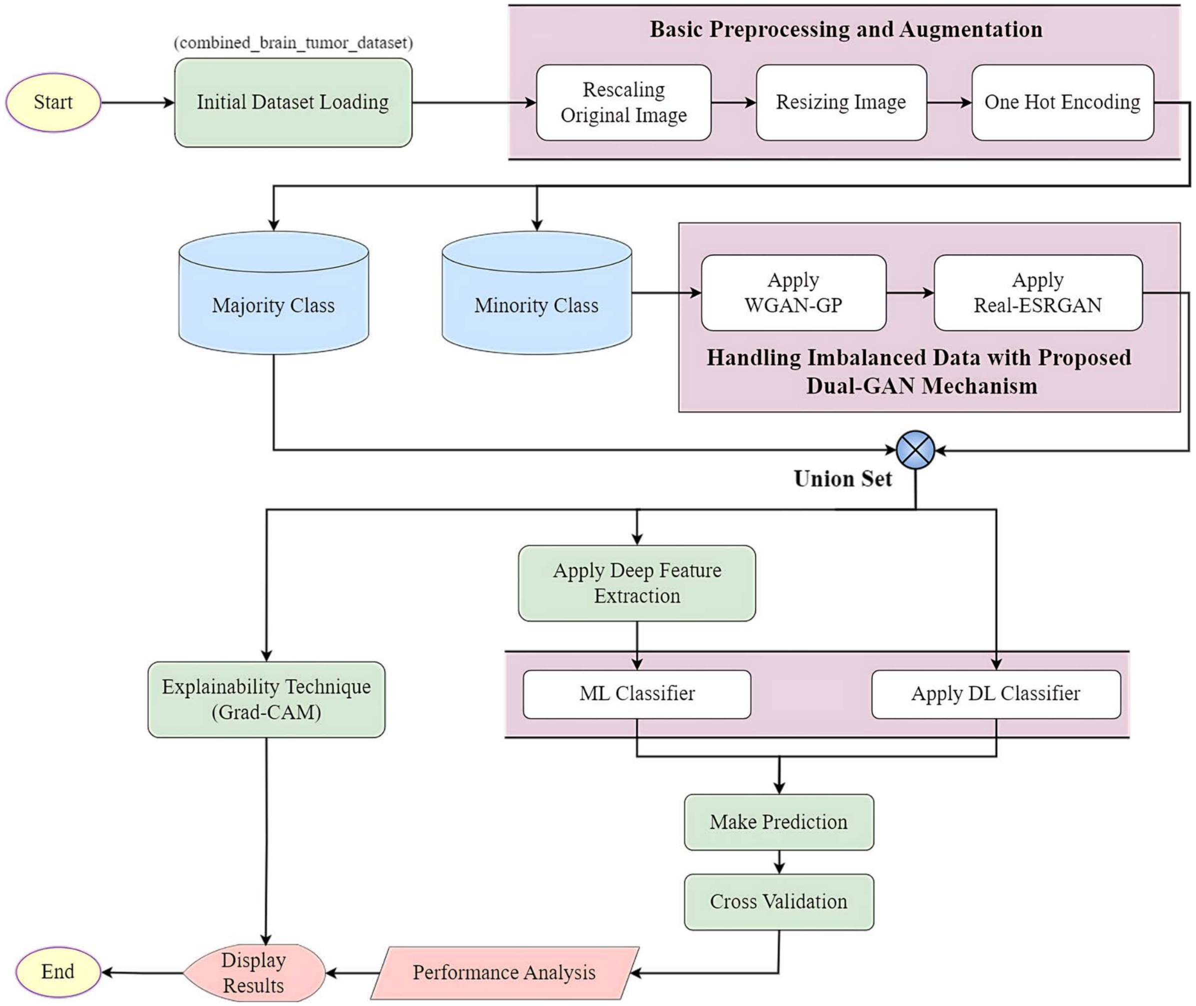


**Fig 2. Flowchart of the proposed methodology.**

https://doi.org/10.1371/journal.pone.0310748.g002

## Dataset description and preprocessing

There are a hundred different distinct classes of brain tumors. However, most researches address either binary classification or only three to four classes. In the process, those studies failed to address more brain tumor types as well as the data imbalance issue. Therefore, we have collected samples from different datasets to include more brain tumor types and to create an imbalanced dataset with classes having different sample sizes.

Dataset one (DS-I) is the combined version of Figshare, SARTAJ dataset, and Br35H [37]. The Figshare dataset encompasses 3,064 MRI images captured in the T1-weighted contrast-enhanced mode. These images collectively represent three distinct categories of brain tumors, with the following distribution: meningioma, glioma, and pituitary tumors. More images on these classes and also for the 'no_tumor' class were collected from the other two datasets. Collectively, we gathered 7,023 images of human brain MRI images of four classes: 'glioma', 'meningioma', 'no_tumor', and 'pituitary' from these three sources. From, the second dataset (DS-II), we have collected 70 samples for 'craniopharyngioma' [38]. The total no. of MRI images used is 7,103 where the 'glioma', the 'meningioma', the 'no_tumor', the 'pituitary', and the 'craniopharyngioma' classes contain 1621, 1645, 2000, 1767 and 70 images respectively. A snapshot of the utilized dataset is presented in Fig 3. It further depicts the imbalance nature of the dataset.

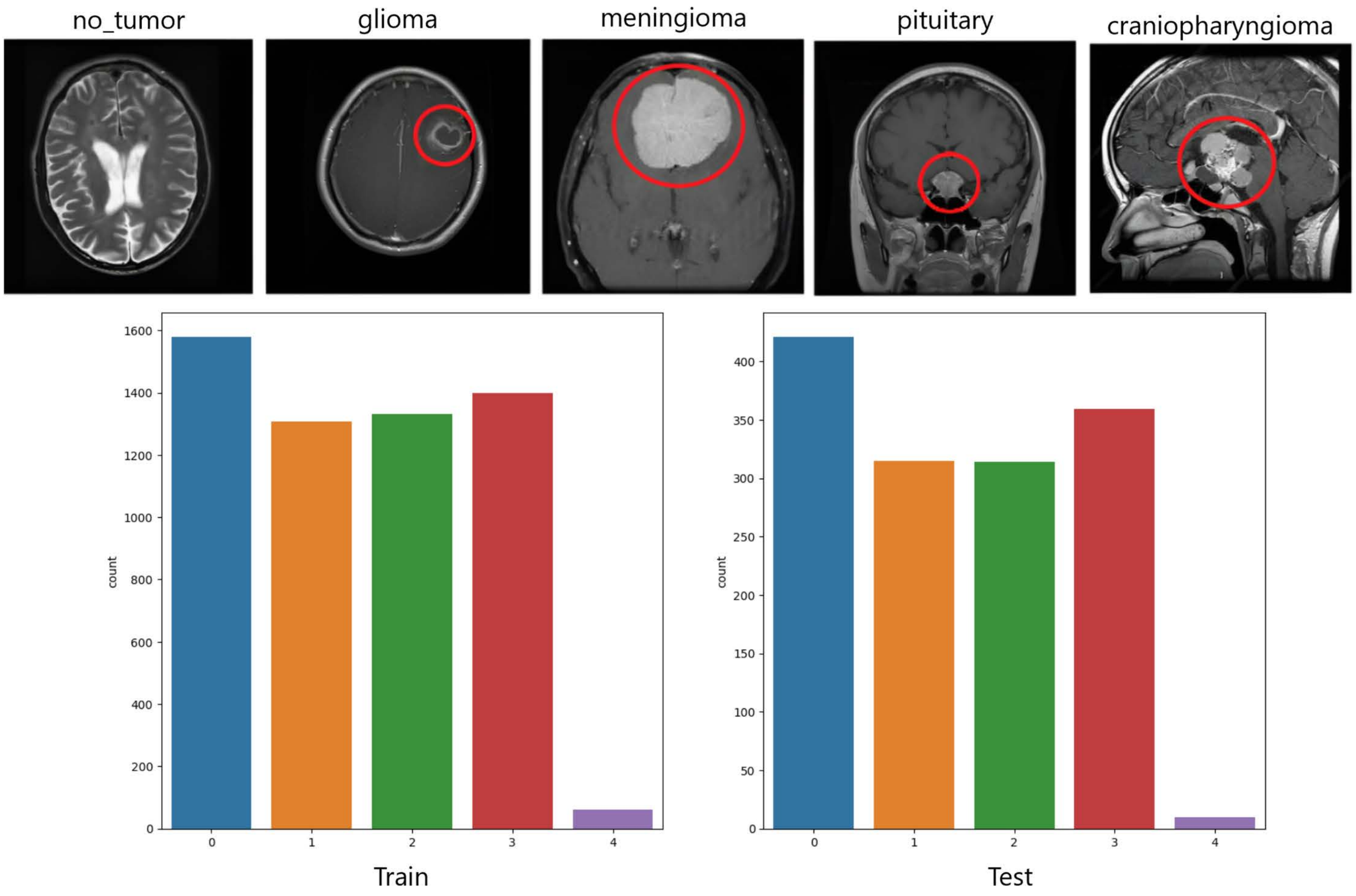


**Fig 3. Dataset samples and its imbalanced nature.**

https://doi.org/10.1371/journal.pone.0310748.g003

We utilize one hot encoding to convert categorical variables in the datasets into numerical representations, enabling us to incorporate them as model-trainable features. Since the dataset does not have any ordinal rank between the target classes, this encoding technique allowed to maintain the integrity of the categorical information while enabling models to process the data effectively. To maintain input consistency and enhance model training, we implemented pixel value normalization on the images. Additionally, the normalization process helps to ensure that each feature contributes equally to the learning process, preventing any particular feature from dominating the others. This can lead to more stable and efficient model training, ultimately resulting in better performance and generalization to new data. Finally, a variety of data augmentation techniques were implemented, including zooming, cropping, rotation, and resizing. We resized all images to a uniform size of 240 × 240.

## Proposed Dual-GAN mechanism

Generative Adversarial Networks (GANs) are a well-known subset of Deep Adversarial Networks (DANs) that focus on generating synthetic data, such as images, audio, and text, that is convincingly similar to real data. By enabling the generation of synthetic data, they enhance model training and address data imbalance challenges [39].

Wasserstein GAN (WGAN) uses the Wasserstein loss to stabilize the model but it further imposed problems like the vanishing gradient problem and produced poor samples while failing to converge. In 2017, I. Gulrajani et al. introduced a gradient penalty to enhance stability, discouraging gradients with substantial norm values, albeit resulting in extended computational time [40]. This model is commonly referred to as a WGAN-GP model. On the other hand, Enhanced Super-Resolution GANs or ESRGAN is a specialized GAN model used to enhance the resolution of the input image. It improves the image quality by generating super-resolution images from low-quality (resolution) images leading to better classification tasks [41].

Generating high-resolution synthetic data directly with WGAN-GP is computationally intensive and costly. Furthermore, training such a network would have cost a great amount of time and complexity along with requiring sophisticated hardware facilities. In contrast, generating low-resolution synthetic data (only 28 × 28) can reduce the computational cost and burden during the initial synthetic data generation phase. Therefore, to handle the small data size for our minority class 'craniopharyngioma' we propose a hybrid technique which we named the 'Dual-GAN' mechanism. We divided the proposed hybrid mechanism into two phases, 1) Data Generation, and 2) Data Enhancement. The proposed mechanism is shown in Fig 4.

While the first phase provides a better generalization and diversity in generated synthetic data, the next phase enhances the image quality (240 × 240) and ensures fine-tuning of the final output images used for the classification task. Additionally, Real-ESRGAN performs progressive refinement of the low-resolution input images itself to capture the most relevant information and outputs better and more realistic images. Furthermore, because of the optimized architecture of Real-ESRGAN, most calculation is performed in a smaller resolution space, which reduces the GPU memory and computational resources consumption [42]. Algorithm 1 explains the working principles of the proposed Dual-GAN mechanism.

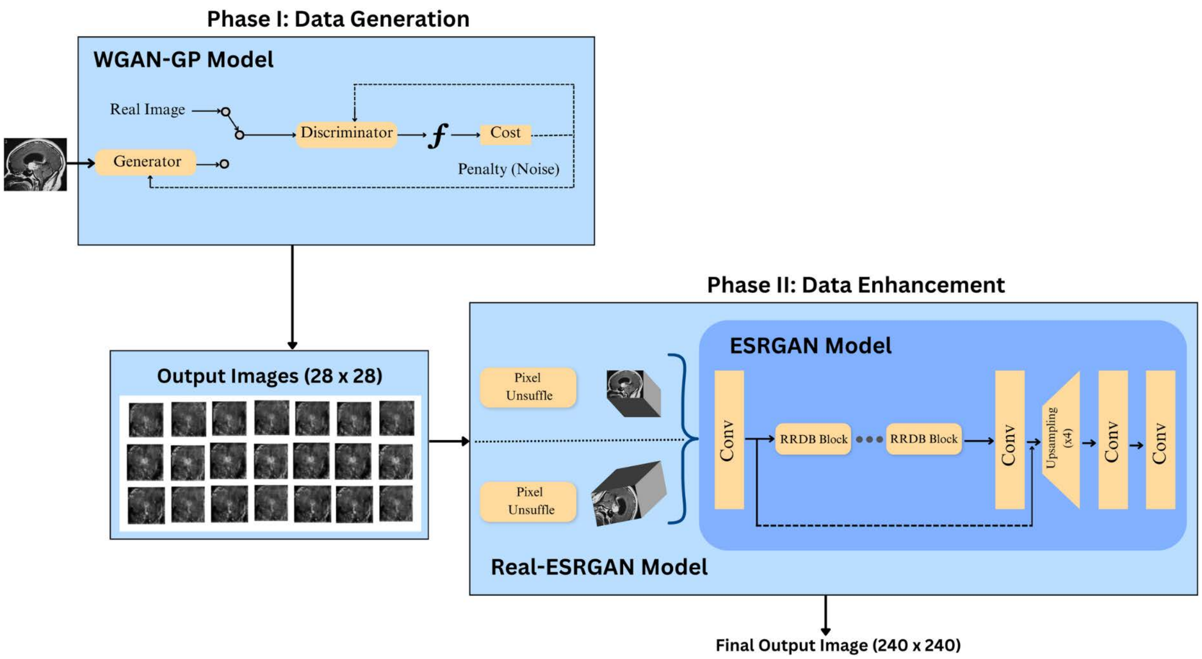


**Fig 4. The proposed dual-GAN mechanism.**

https://doi.org/10.1371/journal.pone.0310748.g004

**Algorithm 1** Proposed Dual-GAN Mechanism

```
Input: WGAN-GP, RealESRGAN, DS1,   // DS1 contains the minority class
       images
EPOCH_NUMBER, BATCH_SIZE
Output: Balanced_Class
Initialization:
Low-res_Data = [ ]     // An empty list to store generated low-res-
olution images
Enhanced_Data = [ ]     // An empty list to store enhanced syn-
thetic images
Balanced_Class = [ ]     // An empty list to store the real and
synthetic images
for each in range(EPOCH_NUMBER)
  for each in range (BATCH_SIZE)
    Low-res_Data = (WGAN-GP, DS1)   // Perform WGAN-GP
    UPDATE WGAN-GP(parameters)
for each in Low-res_Data
  Enhanced_Data = (RealESRGAN, Low-res_Data)   // Perform
  RealESRGAN
Balanced_Class[ ] = Enhanced_Data[ ] || DS1[ ]   // concatenation
operation
return Balanced_Class   // return the class with balanced data
```

## Deep feature extraction

Feature extraction is a fundamental step in our proposed image analysis pipeline, where we aim to extract meaningful and discriminative features from the input data. It involves converting an image into a numerical vector encapsulating its semantic content. These features are further utilized to train the classification models to accurately classify and analyze images based on their content and characteristics.

**VGG16.** The VGG16 architecture, or Visual Geometry Group 16 architecture, was first introduced by Simonyan and Zisserman in 2015 [43]. It is a deep convolutional neural network known for its simplicity and effectiveness. It consists of multiple convolutional layers, followed by max-pooling layers, and culminating in several fully connected layers. VGG16's deep structure allows it to capture intricate features within the images. This hierarchical feature extraction contributes to superior feature representation, making VGG16 a strong choice for image-related tasks. Fig 5 demonstrates the inner layers of the model [44].

**DenseNet201.** DenseNet201, based on the densely connected architecture, is designed to maintain a strong gradient flow and encourage feature reuse across layers [45]. Each layer can access and utilize the feature maps generated by its predecessors. This dense connectivity leads to efficient feature extraction, as the network can collectively capture both low-level and high-level features. DenseNet201's feature-rich representation makes it well-suited for tasks that require the model to recognize complex patterns and relationships within the data.

**InceptionResNetV2.** InceptionResNetV2 is a complex feature extraction model that integrates concepts from the Inception architecture and residual connections [46]. The Inception part employs multiple filter sizes and operations on the input data in parallel, enabling the model to capture features at various scales. Additionally, residual connections facilitate the flow of gradients and prevent the vanishing gradient problem.

**Proposed DeepEFE model.** This study proposes a novel Deep Ensemble Feature Extraction (DeepEFE) model for selecting and combining the valuable features from the

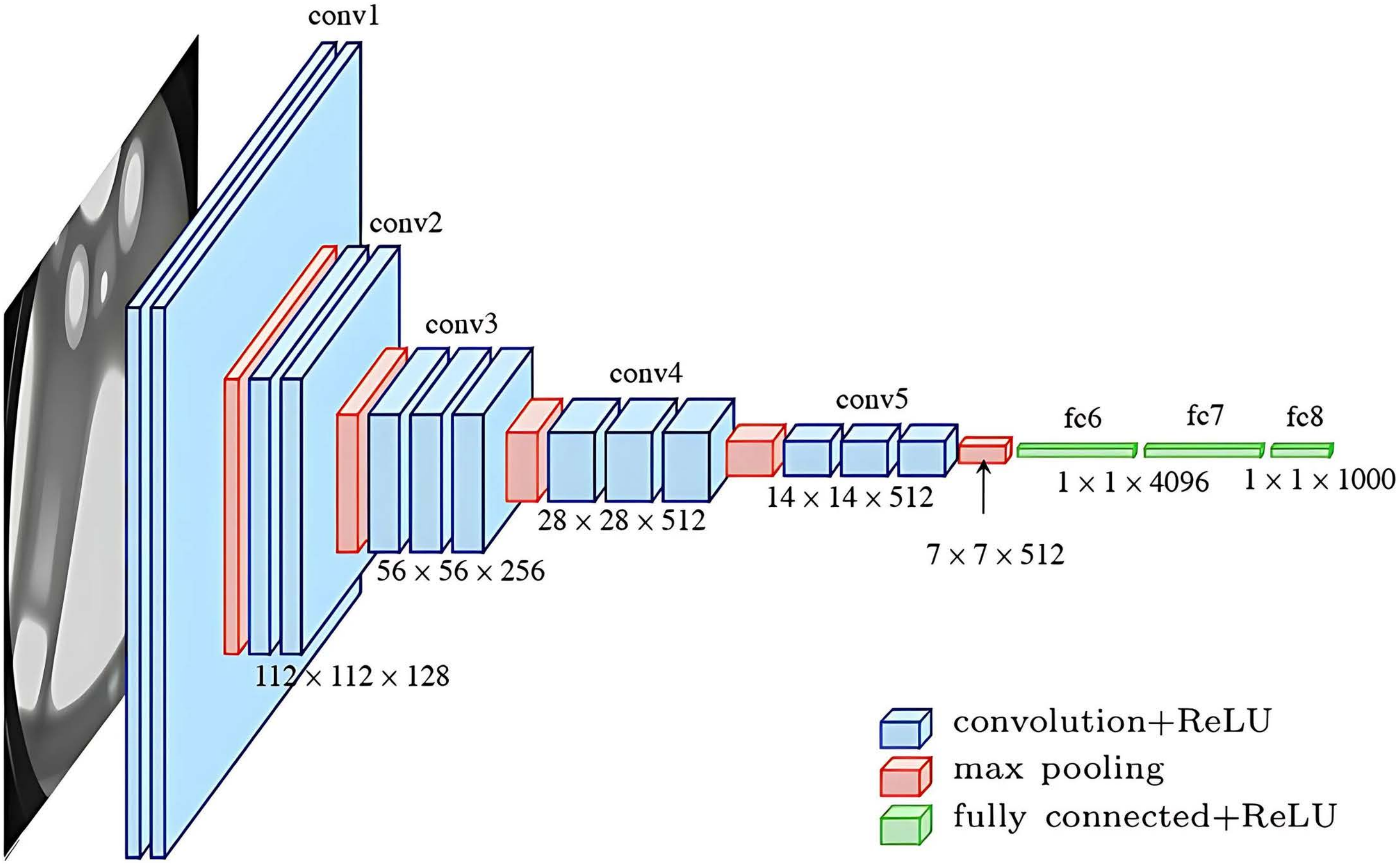


**Fig 5. Architecture of VGG16.**

https://doi.org/10.1371/journal.pone.0310748.g005

input images. It utilizes pre-trained deep learning models like VGG16, DenseNet201, and InceptionResNetV2 to extract rich and diverse features from images of all five classes. The proposed model derives its strength from the enhancements in its model architecture. The individual base models suffer from issues such as increased model complexity, lack of generalization ability, increased training time, and the vanishing gradient problem. However, the proposed DeepEFE effectively addresses these challenges by integrating the benefits from the base models. It maintains optimal performance by consistently ensuring significant accuracy in the image classification task. Our proposed DeepEFE model has the potential to extract a diverse range of rich features using a sophisticated multifaceted feature engineering technique. Furthermore, it is immune to overfitting due to the implementation of densely connected networks, which are specifically designed to efficiently reuse and integrate these extracted features. The deep features are subsequently concatenated into a unified feature vector, thereby enhancing the model's ability to capture subtle and intricate information from the input data. Lastly, this combined vector is fed to the machine learning classifier at the outermost layer for final prediction.

Fig 6 represents the architecture of the DeepEFE model. Here, we can see two major phases which are the Feature Extraction Phase and the Concatenation Phase. The Algorithm 2 further demonstrates the functionalities of the proposed DeepEFE model.

**Algorithm 2** PROPOSED DEEPEFE MODEL

```
Input: Model, Balanced_Dataset    // output dataset from Dual-GAN
Output: Final Extracted Features
Initialization:
1: Final_Features = []        // empty list
2: F1 = FEATURESELECTION(VGG16, Balanced_Dataset)     // returned feature
   set 1
3: F2 = FEATURESELECTION(DenseNet201, Balanced_Dataset)     // returned
   feature set 2
4: F3 = FEATURESELECTION(InceptionResNetV2, Balanced_Dataset)     //
   returned feature set 3
5: Final_Features = F1||F2||F3     // set concatenation operation
6: return Final_Features      // return the final feature list
```

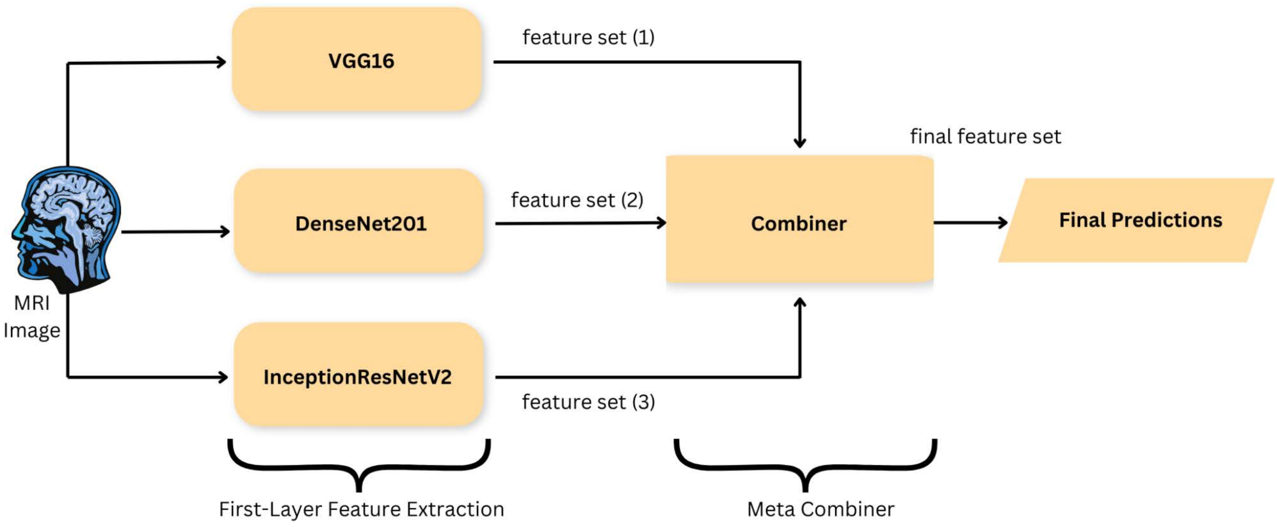


**Fig 6. DeepEFE model architecture.**

https://doi.org/10.1371/journal.pone.0310748.g006

## Description of classification algorithms

*Support Vector Machine (SVM)* is a powerful supervised machine learning algorithm used for classification tasks. It operates by finding the optimal hyperplane that best separates data points in different classes within a high-dimensional feature space. Its core objective is to maximize the margin between classes, making it robust against overfitting. Whereas, *The Random Forest (RF)* classifier is a machine-learning method that builds multiple decision trees during training. Through the bagging technique, it constructs these trees using random subsets of the training data, ensuring diversity among them and reducing overfitting. At each node of the trees, a random subset of features is considered for splitting, adding further randomness. *XGBoost* is another popular classifier algorithm that minimizes an object function to reach the desired outcome.

Unlike traditional neural networks, *CNNs* are designed to automatically and adaptively learn spatial hierarchies of features from input data. At the core of CNNs are convolutional layers, which apply filters (kernels) to the input data to capture local patterns and features. These convolutional operations are followed by pooling layers that downsample the spatial dimensions of the data, reducing computational complexity while preserving important information. *ResNet50*, short for "Residual Network with 50 layers", is built on residual learning. Traditional deep networks aim to learn data's underlying features but can struggle with vanishing gradients in deeper architectures. ResNet50 overcomes this by introducing residual blocks with skip connections, enabling the network to learn residual functions directly. We have explored an *Ensemble Deep Learning (EDL)* model created by averaging the predictions of VGG16, InceptionResNetV2, and DenseNet201. The model architecture of the ensemble model is illustrated in Table 2. This approach harnesses the strengths of each model to enhance the overall performance as it integrates the feature extraction and classification capabilities of VGG16 and includes the capacity of DenseNet to capture hierarchical features from the input images.

## Performance metrics

One commonly used performance metric is *accuracy*, which measures the overall correctness of the model's predictions. *Precision* is a performance metric that focuses on the model's ability to correctly identify negative instances. *Sensitivity*, also known as the recall rate or true positive rate, quantifies the proportion of true positives to the total number of actual positive instances in the dataset. The *F1-Score* is a balanced measure that combines precision and sensitivity into a single metric. *Area under the ROC Curve (AUC)* represents the ability of the

**Table 2. Architecture of Ensemble Deep Learning (EDL) Model.**

| Layer (type) | Output Shape | Param, # | Connected to |
|---|---|---|---|
| input_7 (InputLayer) | [(None,240, 3)] | 0 | [] |
| model (Functional) | (None, 5) | 44,270,597 | ['input_7[0][0]'] |
| model_1 (Functional) | (None, 5) | 44,270,597 | ['input_7[0][0]'] |
| model_2 (Functional) | (None, 5) | 68,526,309 | ['input_7[0][0]' |
| average (Average) | (None, 5) | 0 | model[0][0]<br>model_1[0][0]<br>model_2[0][0] |
| **Total params:**<br>**Trainable params:**<br>**Non-trainable params:** | 157,067,503<br>102,730,767<br>54,336,736 | | |

The above table denotes the architecture of a hybrid ensemble deep learning (EDL) model introduced in this study. This EDL model is a combination of all three of the, InceptionResNetV2, and DenseNet201.

https://doi.org/10.1371/journal.pone.0310748.t002

model to discriminate between positive and negative classes across various threshold values. *False Positive Rate (FPR)* quantifies the proportion of actual negative cases that were incorrectly classified as positive by the model. *False Negative Rate (FNR)* quantifies the proportion of actual negative cases that were incorrectly classified as negative by the model. *True-negative rate (TNR)*, also known as Specificity, measures the proportion of actual negative cases that are correctly identified by a binary classification model.

$$\text{Accuracy} = \frac{\text{TP} + \text{TN}}{\text{TP} + \text{TN} + \text{FP} + \text{FN}} \tag{1}$$

$$\text{Precision} = \frac{\text{TP}}{\text{TP} + \text{FP}} \tag{2}$$

$$\text{Sensitivity} = \frac{\text{TP}}{\text{TP} + \text{FN}} \tag{3}$$

$$\text{F1-Score} = 2 \bullet \frac{\text{Precision} \bullet \text{Sensitivity}}{\text{Precision} + \text{Sensitivity}} \tag{4}$$

$$AUC = \int_0^1 \text{TPR}(\text{FPR})\, d\text{FPR} \tag{5}$$

$$\text{FPR} = \frac{\text{FP}}{\text{FP} + \text{TN}} \tag{6}$$

$$\text{FNR} = \frac{\text{FN}}{\text{FN} + \text{TN}} \tag{7}$$

$$\text{Specificity} = \frac{\text{TN}}{\text{TN} + \text{FP}} \tag{8}$$

where TP, TN, FP, and FN refer to True Positive, True Negative, False Positive, and False Negative, respectively.

*Training Accuracy* is a performance metric that measures the model's ability to predict the training dataset's labels or outcomes correctly. High training accuracy indicates that the model can accurately predict examples it has seen during training. *Validation Accuracy* assesses the model's predictive performance on a separate dataset called the validation dataset. A *Loss Curve*, also known as a learning curve, tracks the model's loss (a measure of error) on both the training and validation datasets across multiple training iterations or epochs. To evaluate the efficiency of the proposed Dual-GAN mechanism, generator and discriminator loss curves were assessed.

## Experimental results and discussions

This Experimental Results and Discussions section demonstrates the experimental results of all the stages of this research and provides a detailed analysis of those outcomes. The coding files of our proposed pipeline and experimental outcomes are available at https://github.com/roypriyanka7/Brain-Tumor-Classification.

## Hyperparameter settings for the models used

Hyperparameter setup involves the formal definition of the configuration and values of hyperparameters for an ML learning model. These parameters control the behaviour of the deep learning models and guide the learning process as well. Table 3 illustrates the parameters for each algorithm in the proposed pipeline. These parameters are crucial for optimizing model performance and its generalizability.

## Effect of imbalanced dataset on model prediction

This study aims to show the effect of class imbalance on a certain model's classification predictions. Our investigation commenced with an examination of the impact of an imbalanced dataset on brain tumor classification. As expected, the class imbalance introduced challenges in correctly identifying and distinguishing between tumor types. Table 4 represents the performance of our classification models on the image dataset before applying the proposed Dual-GAN mechanism.

As the table shows, the proposed deep feature-based DeepEFE model surpassed all other models by achieving an accuracy of 97.26% on the imbalanced dataset. Yet a prime concern is that, although these models performed well for no_tumor, glioma, meningioma and pituitary classes (majority classes), the model performance for the minority class 'craniopharyngioma' is not satisfactory. The sensitivity value for the minority class is alarming (only 42%). This recall value of 42% indicates that the model can not even predict half of the positive cases correctly. This means that the model's performance is too poor and unreliable, significantly in the case of highly sensitive medical data where missing cases can have serious consequences. Fig 7 and Fig 8 provide more evidence regarding this scenario. Especially, the purple line depicting the minority class is seen as having lower values both in the AUC curve and the precision-recall curve.

## Performance after applying proposed methodology

Mitigating the issue of imbalanced datasets in the medical field is crucial because it directly impacts patient care and diagnostic accuracy. Class imbalance can lead to biased model

**Table 3. Hyperparameter settings for the models used.**

| Model | Hyperparameter(s) |
|---|---|
| CNN | no. of layers = 7, optimizer = 'adam', epochs = [40, 40], loss_function = sparse_categorical_crossentropy, activation = ReLU, metrics = ['accuracy'] |
| ResNet50 | weights = 'imagenet', optimizer = 'adam', epochs = [45, 42], loss_function = categorical_crossentropy, include_top = False, metrics = ['accuracy'] |
| EDL | optimizer = adam, learning_rate = 0.0001, loss_function = sparse_categorical_crossentropy, metrics = ['accuracy'], include_top = False, epochs = [45, 27] |
| VGG16 | weights = 'imagenet', optimizer = 'adam', include_top = False, classifier_activation = softmax |
| Inception-ResNetV2 | weights = 'imagenet', include_top = False, classifier_activation = softmax |
| DenseNet201 | weights = 'imagenet', include_top = False, classifier_activation = softmax |
| DeepEFE | Base Models: DenseNet201, InceptionResNetV2, VGG16<br>weights: 'imagenet', optimizer = 'adam', include_top = False, classifier_activation = softmax<br>Concatenation: Features from DenseNet201, InceptionResNetV2, and VGG16 |
| SVM | kernel = 'linear', probability = True |
| RF | n_estimators = 100, random_state = 42 |
| XGB | max_depth = 3, learning_rate = 0.1, n_estimators = 100 |

This table presents the experimental setup and optimized hyperparameters for each of the utilized algorithms. The parameter `epoch' represents the number of epochs needed for a model to converge and make the final predictions.

https://doi.org/10.1371/journal.pone.0310748.t003

**Table 4. Performance of the classification models before applying dual-GAN.**

| Model | Class | Precision | Sensitivity | F1-Score | Accuracy (%) | FPR | FNR | Specificity |
|---|---|---|---|---|---|---|---|---|
| **State-of-the-Art Deep Learning Models** | | | | | | | | |
| **CNN** | no_tumor | 0.98 | 0.97 | 0.97 | | 0.01 | 0.03 | 0.99 |
| | glioma | 0.96 | 0.95 | 0.95 | | 0.01 | 0.05 | 0.99 |
| | meningioma | 0.92 | 0.94 | 0.93 | 95.71 | 0.02 | 0.06 | 0.98 |
| | pituitary | 0.97 | 0.99 | 0.98 | | 0.01 | 0.01 | 0.99 |
| | craniopharyngioma | 0.40 | 0.17 | 0.24 | | 0.00 | 0.83 | 1.00 |
| **ResNet50** | no_tumor | 0.93 | 0.96 | 0.94 | | 0.03 | 0.04 | 0.97 |
| | glioma | 0.98 | 0.88 | 0.93 | | 0.00 | 0.12 | 1.00 |
| | meningioma | 0.85 | 0.94 | 0.89 | 92.19 | 0.05 | 0.06 | 0.95 |
| | pituitary | 0.96 | 0.93 | 0.94 | | 0.01 | 0.07 | 0.99 |
| | craniopharyngioma | 0.25 | 0.25 | 0.25 | | 0.01 | 0.75 | 0.99 |
| **EDL** | no_tumor | 0.95 | 0.97 | 0.96 | | 0.02 | 0.03 | 0.98 |
| | glioma | 0.93 | 0.90 | 0.91 | | 0.02 | 0.10 | 0.98 |
| | meningioma | 0.80 | 0.80 | 0.80 | 90.36 | 0.06 | 0.20 | 0.94 |
| | pituitary | 0.92 | 0.95 | 0.93 | | 0.03 | 0.05 | 0.97 |
| | craniopharyngioma | 0.00 | 0.00 | 0.00 | | 0.00 | 1.00 | 1.00 |
| **Deep Feature Extraction** | | | | | | | | |
| **VGG16 + SVM** | no_tumor | 0.99 | 0.98 | 0.99 | | 0.00 | 0.02 | 1.00 |
| | glioma | 0.98 | 0.96 | 0.97 | | 0.01 | 0.04 | 0.99 |
| | meningioma | 0.92 | 0.98 | 0.95 | 96.83 | 0.03 | 0.02 | 0.97 |
| | pituitary | 0.98 | 0.98 | 0.98 | | 0.01 | 0.02 | 0.99 |
| | craniopharyngioma | 0.60 | 0.25 | 0.35 | | 0.00 | 0.75 | 1.00 |
| **VGG16 + RF** | no_tumor | 0.97 | 0.96 | 0.97 | | 0.01 | 0.04 | 0.98 |
| | glioma | 0.96 | 0.88 | 0.92 | | 0.01 | 0.12 | 0.98 |
| | meningioma | 0.85 | 0.88 | 0.87 | 91.84 | 0.04 | 0.12 | 0.94 |
| | pituitary | 0.88 | 0.98 | 0.93 | | 0.04 | 0.02 | 0.94 |
| | craniopharyngioma | 0.00 | 0.00 | 0.00 | | 0.00 | 1.00 | 1.00 |
| **VGG16 + XGB** | no_tumor | 0.98 | 0.97 | 0.97 | | 0.01 | 0.03 | 0.98 |
| | glioma | 0.97 | 0.93 | 0.95 | | 0.01 | 0.07 | 0.99 |
| | meningioma | 0.89 | 0.92 | 0.91 | 94.51 | 0.03 | 0.08 | 0.96 |
| | pituitary | 0.94 | 0.98 | 0.96 | | 0.02 | 0.02 | 0.97 |
| | craniopharyngioma | 1.00 | 0.08 | 0.15 | | 0.00 | 0.92 | 1.00 |
| **Inception-ResNetV2 + SVM** | no_tumor | 0.99 | 0.99 | 0.99 | | 0.00 | 0.01 | 1.00 |
| | glioma | 0.96 | 0.94 | 0.95 | | 0.01 | 0.06 | 0.99 |
| | meningioma | 0.89 | 0.92 | 0.91 | 95.43 | 0.03 | 0.08 | 0.97 |
| | pituitary | 0.96 | 0.98 | 0.97 | | 0.01 | 0.02 | 0.99 |
| | craniopharyngioma | 1.00 | 0.42 | 0.59 | | 0.00 | 0.58 | 1.00 |
| **Inception-ResNetV2 + RF** | no_tumor | 0.98 | 0.96 | 0.97 | | 0.01 | 0.04 | 0.99 |
| | glioma | 0.90 | 0.84 | 0.87 | | 0.03 | 0.16 | 0.97 |
| | meningioma | 0.82 | 0.82 | 0.82 | 89.3 | 0.05 | 0.18 | 0.95 |
| | pituitary | 0.86 | 0.97 | 0.91 | | 0.05 | 0.03 | 0.95 |
| | craniopharyngioma | 0.00 | 0.00 | 0.00 | | 0.00 | 1.00 | 1.00 |
| **Inception-ResNetV2 + XGB** | no_tumor | 0.98 | 0.97 | 0.98 | | 0.01 | 0.03 | 0.99 |
| | glioma | 0.95 | 0.88 | 0.91 | | 0.01 | 0.12 | 0.99 |
| | meningioma | 0.82 | 0.88 | 0.85 | 91.91 | 0.05 | 0.12 | 0.95 |
| | pituitary | 0.91 | 0.97 | 0.94 | | 0.03 | 0.03 | 0.97 |
| | craniopharyngioma | 0.00 | 0.00 | 0.00 | | 0.00 | 1.00 | 1.00 |

*(Continued)*

Table 4. (Continued)

| Model | Class | Precision | Sensitivity | F1-Score | Accuracy (%) | FPR | FNR | Specificity |
|---|---|---|---|---|---|---|---|---|
| **Dense-Net201 + SVM** | no_tumor | 0.99 | 0.99 | 0.99 | | 0.00 | 0.01 | 1.00 |
| | glioma | 0.97 | 0.97 | 0.97 | | 0.01 | 0.03 | 0.99 |
| | meningioma | 0.92 | 0.96 | 0.94 | 96.97 | 0.02 | 0.04 | 0.98 |
| | pituitary | 0.99 | 0.98 | 0.99 | | 0.00 | 0.02 | 1.00 |
| | craniopharyngioma | 1.00 | 0.33 | 0.50 | | 0.00 | 0.67 | 1.00 |
| **Dense-Net201 + RF** | no_tumor | 0.98 | 0.97 | 0.97 | | 0.01 | 0.03 | 0.99 |
| | glioma | 0.97 | 0.87 | 0.92 | | 0.01 | 0.13 | 0.99 |
| | meningioma | 0.84 | 0.90 | 0.87 | 92.12 | 0.05 | 0.10 | 0.95 |
| | pituitary | 0.89 | 0.96 | 0.93 | | 0.04 | 0.04 | 0.96 |
| | craniopharyngioma | 0.00 | 0.00 | 0.00 | | 0.00 | 1.00 | 1.00 |
| **Dense-Net201 + XGB** | no_tumor | 0.97 | 0.99 | 0.98 | | 0.01 | 0.01 | 0.99 |
| | glioma | 0.99 | 0.93 | 0.96 | | 0.00 | 0.07 | 1.00 |
| | meningioma | 0.90 | 0.92 | 0.91 | 95.21 | 0.03 | 0.08 | 0.97 |
| | pituitary | 0.94 | 0.99 | 0.96 | | 0.02 | 0.01 | 0.98 |
| | craniopharyngioma | 1.00 | 0.08 | 0.15 | | 0.00 | 0.92 | 1.00 |
| **DeepEFE + SVM** | no_tumor | 0.99 | 0.99 | 0.99 | | 0.00 | 0.01 | 1.00 |
| | glioma | 0.98 | 0.97 | 0.98 | | 0.01 | 0.03 | 0.99 |
| | meningioma | 0.93 | 0.96 | 0.94 | 97.26 | 0.02 | 0.04 | 0.98 |
| | pituitary | 0.98 | 0.98 | 0.98 | | 0.01 | 0.02 | 0.99 |
| | craniopharyngioma | 0.83 | 0.42 | 0.56 | | 0.00 | 0.58 | 1.00 |
| **DeepEFE + RF** | no_tumor | 0.98 | 0.97 | 0.98 | | 0.01 | 0.03 | 0.99 |
| | glioma | 0.97 | 0.88 | 0.92 | | 0.01 | 0.12 | 0.99 |
| | meningioma | 0.84 | 0.87 | 0.85 | 91.91 | 0.05 | 0.13 | 0.95 |
| | pituitary | 0.88 | 0.97 | 0.92 | | 0.04 | 0.03 | 0.96 |
| | craniopharyngioma | 0.00 | 0.00 | 0.00 | | 0.00 | 1.00 | 1.00 |
| DeepEFE + XGB | no_tumor | 0.98 | 0.98 | 0.98 | | 0.01 | 0.02 | 0.99 |
| | glioma | 0.98 | 0.92 | 0.95 | | 0.00 | 0.08 | 1.00 |
| | meningioma | 0.88 | 0.93 | 0.90 | 94.58 | 0.04 | 0.07 | 0.96 |
| | pituitary | 0.94 | 0.98 | 0.96 | | 0.02 | 0.02 | 0.98 |
| | craniopharyngioma | 0.75 | 0.25 | 0.38 | | 0.00 | 0.75 | 1.00 |

This table illustrates the outcomes of different models during the imbalanced dataset. The performance of the minority class is very poor in comparison with the other majority classes, which further contributed to the overall poor outcome of the models.

https://doi.org/10.1371/journal.pone.0310748.t004

predictions, particularly in scenarios where the minority class represents critical medical conditions. In our case, it represents a whole group of brain tumor categories, and as evident in Table 4, class imbalance significantly decreased the model's reliability and prediction accuracy in terms of distinguishing minority classes from majority classes. Addressing class imbalance ensures that machine learning models provide a more balanced and reliable assessment, leading to early detection and improved patient outcomes.

To palliate the imbalance dataset issue, we leveraged the power of Generative Adversarial Networks (GANs) and generated synthetic samples for the minority class. This approach was a strategic choice, as it aimed to address the class imbalance by artificially increasing the representation of the minority class, namely 'craniopharyngioma'. By introducing additional synthetic samples of this minority class, the model gains a better understanding of the unique features associated with it. Table 5 provides the results of classification models after applying our proposed Dual-GAN mechanism. This table showcases the positive impact of our

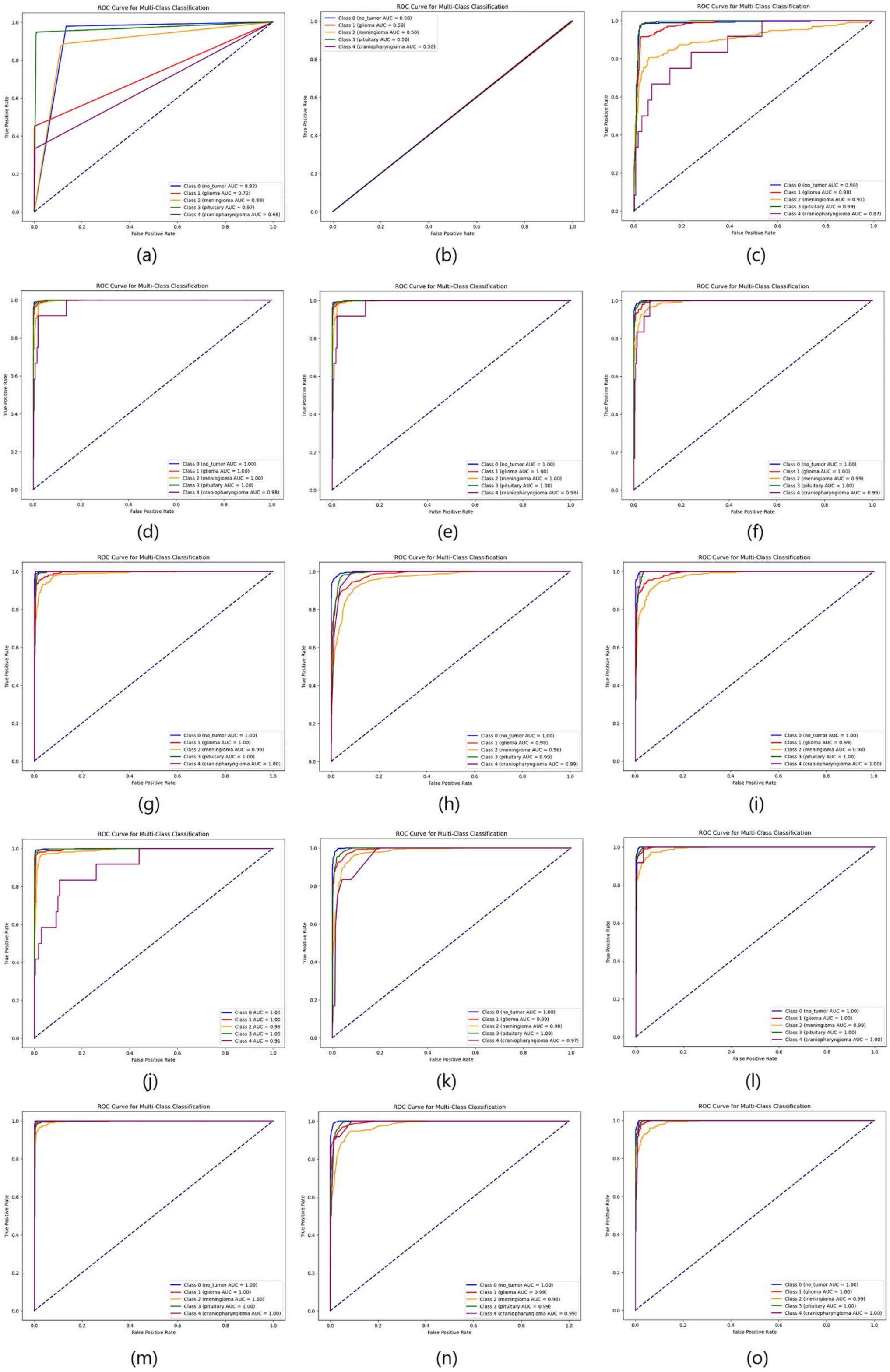

**Fig 7. Comparison of AUC curves on imbalanced dataset.** (a) CNN, (b) ResNet50, (c) EDL, (d) VGG16 + SVM, (e) VGG16 + RF, (f) VGG16 + XGB, (g) InceptionResnetV2 + SVM, (h) InceptionResnetV2 + RF, (i) InceptionResnetV2 + XGB, (j) DenseNet201 + SVM, (k) DenseNet201 + RF, (l) DenseNet201 + XGB, (m) DeepEFE + SVM, (n) DeepEFE + RF, (o) DeepEFE + XGB.

https://doi.org/10.1371/journal.pone.0310748.g007

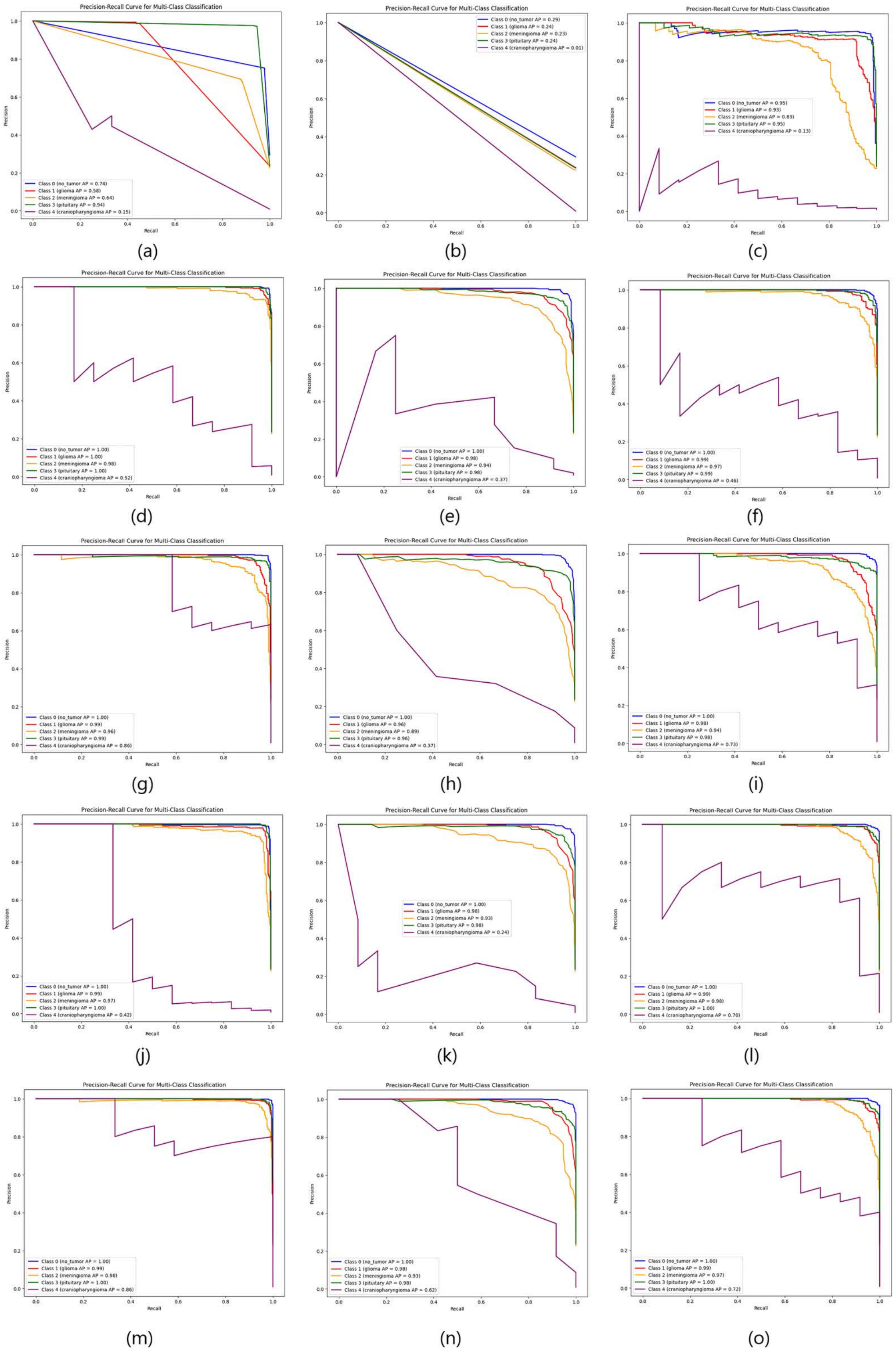


**Fig 8. Comparison of Precision-recall curves for imbalanced dataset.** (a) CNN, (b) ResNet50, (c) EDL, (d) VGG16 + SVM, (e) VGG16 + RF, (f) VGG16 + XGB, (g) InceptionResnetV2 + SVM, (h) InceptionResnetV2 + RF, (i) InceptionResnetV2 + XGB, (j) DenseNet201 + SVM, (k) DenseNet201 + RF, (l) DenseNet201 + XGB, (m) DeepEFE + SVM, (n) DeepEFE + RF, (o) DeepEFE + XGB.

https://doi.org/10.1371/journal.pone.0310748.g008

**Table 5. Performance of Deep Feature Extraction Models after Applying Dual-GAN.**

| Model | Class | Precision | Sensitivity | F1-Score | Accuracy (%) | FPR | FNR | Specificity |
|---|---|---|---|---|---|---|---|---|
| **State-of-the-Art Deep Learning Models** | | | | | | | | |
| **CNN** | no_tumor | 0.98 | 0.97 | 0.97 | | 0.01 | 0.03 | 0.99 |
| | glioma | 0.96 | 0.93 | 0.95 | | 0.01 | 0.05 | 0.99 |
| | meningioma | 0.91 | 0.94 | 0.92 | 95.99 | 0.02 | 0.06 | 0.98 |
| | pituitary | 0.98 | 0.99 | 0.98 | | 0.01 | 0.01 | 0.99 |
| | craniopharyngioma | 0.98 | 0.97 | 0.98 | | 0.00 | 0.83 | 1.00 |
| **ResNet50** | no_tumor | 0.98 | 0.94 | 0.96 | | 0.01 | 0.06 | 0.99 |
| | glioma | 0.92 | 0.91 | 0.92 | | 0.02 | 0.09 | 0.98 |
| | meningioma | 0.84 | 0.88 | 0.86 | 93.28 | 0.05 | 0.12 | 0.95 |
| | pituitary | 0.96 | 0.97 | 0.97 | | 0.01 | 0.03 | 0.99 |
| | craniopharyngioma | 0.99 | 0.96 | 0.98 | | 0.00 | 0.04 | 1.00 |
| **EDL** | no_tumor | 0.95 | 0.95 | 0.95 | | 0.02 | 0.05 | 0.98 |
| | glioma | 0.91 | 0.89 | 0.9 | | 0.02 | 0.11 | 0.98 |
| | meningioma | 0.84 | 0.76 | 0.8 | 91.12 | 0.04 | 0.24 | 0.96 |
| | pituitary | 0.88 | 1.00 | 0.93 | | 0.04 | 0.00 | 0.96 |
| | craniopharyngioma | 0.99 | 0.97 | 0.98 | | 0.00 | 0.03 | 1.00 |
| **Deep Feature Extraction** | | | | | | | | |
| **VGG16 + SVM** | no_tumor | 0.99 | 0.98 | 0.98 | | 0.00 | 0.02 | 1.00 |
| | glioma | 0.98 | 0.96 | 0.97 | | 0.01 | 0.04 | 0.99 |
| | meningioma | 0.93 | 0.96 | 0.95 | 97.29 | 0.02 | 0.04 | 0.98 |
| | pituitary | 0.99 | 0.99 | 0.99 | | 0.00 | 0.01 | 1.00 |
| | craniopharyngioma | 0.99 | 0.97 | 0.98 | | 0.00 | 0.03 | 1.00 |
| **VGG16 + RF** | no_tumor | 0.97 | 0.96 | 0.97 | | 0.01 | 0.04 | 0.99 |
| | glioma | 0.94 | 0.87 | 0.91 | | 0.01 | 0.13 | 0.99 |
| | meningioma | 0.85 | 0.87 | 0.86 | 92.53 | 0.04 | 0.13 | 0.96 |
| | pituitary | 0.89 | 0.97 | 0.93 | | 0.03 | 0.03 | 0.97 |
| | craniopharyngioma | 1.00 | 0.96 | 0.98 | | 0.00 | 0.04 | 1.00 |
| **VGG16 + XGB** | no_tumor | 0.98 | 0.98 | 0.98 | | 0.01 | 0.02 | 0.99 |
| | glioma | 0.97 | 0.91 | 0.94 | | 0.01 | 0.09 | 0.99 |
| | meningioma | 0.88 | 0.9 | 0.89 | 94.69 | 0.03 | 0.10 | 0.97 |
| | pituitary | 0.93 | 0.98 | 0.95 | | 0.02 | 0.02 | 0.98 |
| | craniopharyngioma | 1.00 | 0.96 | 0.98 | | 0.00 | 0.04 | 1.00 |
| **Inception-ResNetV2 + SVM** | no_tumor | 0.99 | 0.98 | 0.99 | | 0.00 | 0.02 | 1.00 |
| | glioma | 0.97 | 0.91 | 0.94 | | 0.01 | 0.09 | 0.99 |
| | meningioma | 0.89 | 0.95 | 0.92 | 96.05 | 0.03 | 0.05 | 0.97 |
| | pituitary | 0.97 | 0.98 | 0.98 | | 0.01 | 0.02 | 0.99 |
| | craniopharyngioma | 0.99 | 0.99 | 0.99 | | 0.00 | 0.01 | 1.00 |
| **Inception-ResNetV2 + RF** | no_tumor | 0.98 | 0.94 | 0.96 | | 0.01 | 0.06 | 0.99 |
| | glioma | 0.94 | 0.83 | 0.88 | | 0.01 | 0.17 | 0.99 |
| | meningioma | 0.80 | 0.82 | 0.81 | 90.07 | 0.05 | 0.18 | 0.95 |
| | pituitary | 0.83 | 0.97 | 0.89 | | 0.05 | 0.03 | 0.95 |
| | craniopharyngioma | 1.00 | 0.96 | 0.98 | | 0.00 | 0.04 | 1.00 |
| **Inception-ResNetV2 + XGB** | no_tumor | 0.99 | 0.96 | 0.97 | | 0.00 | 0.04 | 1.00 |
| | glioma | 0.97 | 0.87 | 0.92 | | 0.01 | 0.13 | 0.99 |
| | meningioma | 0.84 | 0.87 | 0.85 | 92.78 | 0.04 | 0.13 | 0.96 |
| | pituitary | 0.88 | 0.98 | 0.93 | | 0.04 | 0.02 | 0.96 |
| | craniopharyngioma | 1.00 | 0.97 | 0.99 | | 0.00 | 0.03 | 1.00 |

*(Continued)*

**Table 5.** (Continued)

| Model | Class | Precision | Sensitivity | F1-Score | Accuracy (%) | FPR | FNR | Specificity |
|---|---|---|---|---|---|---|---|---|
| **Dense-Net201 + SVM** | no_tumor | 0.99 | 0.98 | 0.99 | | 0.00 | 0.02 | 1.00 |
| | glioma | 0.98 | 0.95 | 0.96 | | 0.01 | 0.05 | 0.99 |
| | meningioma | 0.91 | 0.97 | 0.94 | 97.04 | 0.02 | 0.03 | 0.98 |
| | pituitary | 1.00 | 0.98 | 0.99 | | 0.00 | 0.02 | 1.00 |
| | craniopharyngioma | 0.98 | 0.97 | 0.97 | | 0.00 | 0.03 | 1.00 |
| **Dense-Net201 + RF** | no_tumor | 0.98 | 0.97 | 0.97 | | 0.01 | 0.03 | 0.99 |
| | glioma | 0.97 | 0.86 | 0.91 | | 0.01 | 0.14 | 0.99 |
| | meningioma | 0.86 | 0.89 | 0.87 | 93.03 | 0.04 | 0.11 | 0.96 |
| | pituitary | 0.88 | 0.97 | 0.93 | | 0.04 | 0.03 | 0.96 |
| | craniopharyngioma | 1.00 | 0.96 | 0.98 | | 0.00 | 0.04 | 1.00 |
| **Dense-Net201 + XGB** | no_tumor | 0.99 | 0.97 | 0.98 | | 0.00 | 0.03 | 1.00 |
| | glioma | 0.98 | 0.92 | 0.95 | | 0.00 | 0.08 | 1.00 |
| | meningioma | 0.90 | 0.94 | 0.92 | 95.81 | 0.03 | 0.06 | 0.97 |
| | pituitary | 0.96 | 0.99 | 0.97 | | 0.01 | 0.01 | 0.99 |
| | craniopharyngioma | 0.98 | 0.97 | 0.98 | | 0.00 | 0.03 | 1.00 |
| **DeepEFE + SVM** | no_tumor | 0.99 | 0.99 | 0.99 | | 0.00 | 0.01 | 1.00 |
| | glioma | 0.99 | 0.96 | 0.98 | | 0.00 | 0.04 | 1.00 |
| | meningioma | 0.94 | 0.98 | 0.96 | 98.15 | 0.02 | 0.02 | 0.98 |
| | pituitary | 1.00 | 0.99 | 0.99 | | 0.00 | 0.01 | 1.00 |
| | craniopharyngioma | 0.99 | 0.98 | 0.99 | | 0.00 | 0.02 | 1.00 |
| **DeepEFE + RF** | no_tumor | 0.97 | 0.96 | 0.97 | | 0.01 | 0.04 | 0.99 |
| | glioma | 0.97 | 0.87 | 0.92 | | 0.01 | 0.13 | 0.99 |
| | meningioma | 0.85 | 0.87 | 0.86 | 92.66 | 0.04 | 0.13 | 0.96 |
| | pituitary | 0.88 | 0.98 | 0.92 | | 0.04 | 0.02 | 0.96 |
| | craniopharyngioma | 1.00 | 0.96 | 0.98 | | 0.00 | 0.04 | 1.00 |
| **DeepEFE + XGB** | no_tumor | 0.99 | 0.98 | 0.98 | | 0.00 | 0.02 | 1.00 |
| | glioma | 0.99 | 0.92 | 0.96 | | 0.00 | 0.08 | 1.00 |
| | meningioma | 0.9 | 0.93 | 0.92 | 95.74 | 0.03 | 0.07 | 0.97 |
| | pituitary | 0.93 | 0.98 | 0.96 | | 0.02 | 0.02 | 0.98 |
| | craniopharyngioma | 0.99 | 0.97 | 0.98 | | 0.00 | 0.03 | 1.00 |

The table demonstrates the outcomes of different models after following the proposed pipeline. A significant improvement is observed compared to the previous outcomes, especially in terms of the minor class 'craniopharyngioma'.

https://doi.org/10.1371/journal.pone.0310748.t005

approach in mitigating the class imbalance issue, resulting in enhanced classification performance across all brain tumor categories.

From the table, we can notice a significant change in outcome compared to the previous one. We observe improved sensitivity, F1-Score, and specificity across all brain tumor classes. The performance table shows that the proposed DeepEFE model combined with SVM surpassed other classifiers with a significant accuracy of 98.15%. This suggests that the Dual-GAN mechanism positively impacted the model's ability to correctly classify brain tumors, contributing to higher accuracy. Furthermore, all the models exhibited a considerable improvement in their performance metrics, most notably in terms of sensitivity and specificity. It is noticeable that all the Precision, Sensitivity, and F1-Score across all five (05) classes are between 94–100%, demonstrating our proposed solution's impactful performance in brain tumor diagnosis. Fig 9 and Fig 10 further validate these findings. Here we see a balanced AUC curve and precision-recall curve, although the performance of ResNet50 and the EDL model

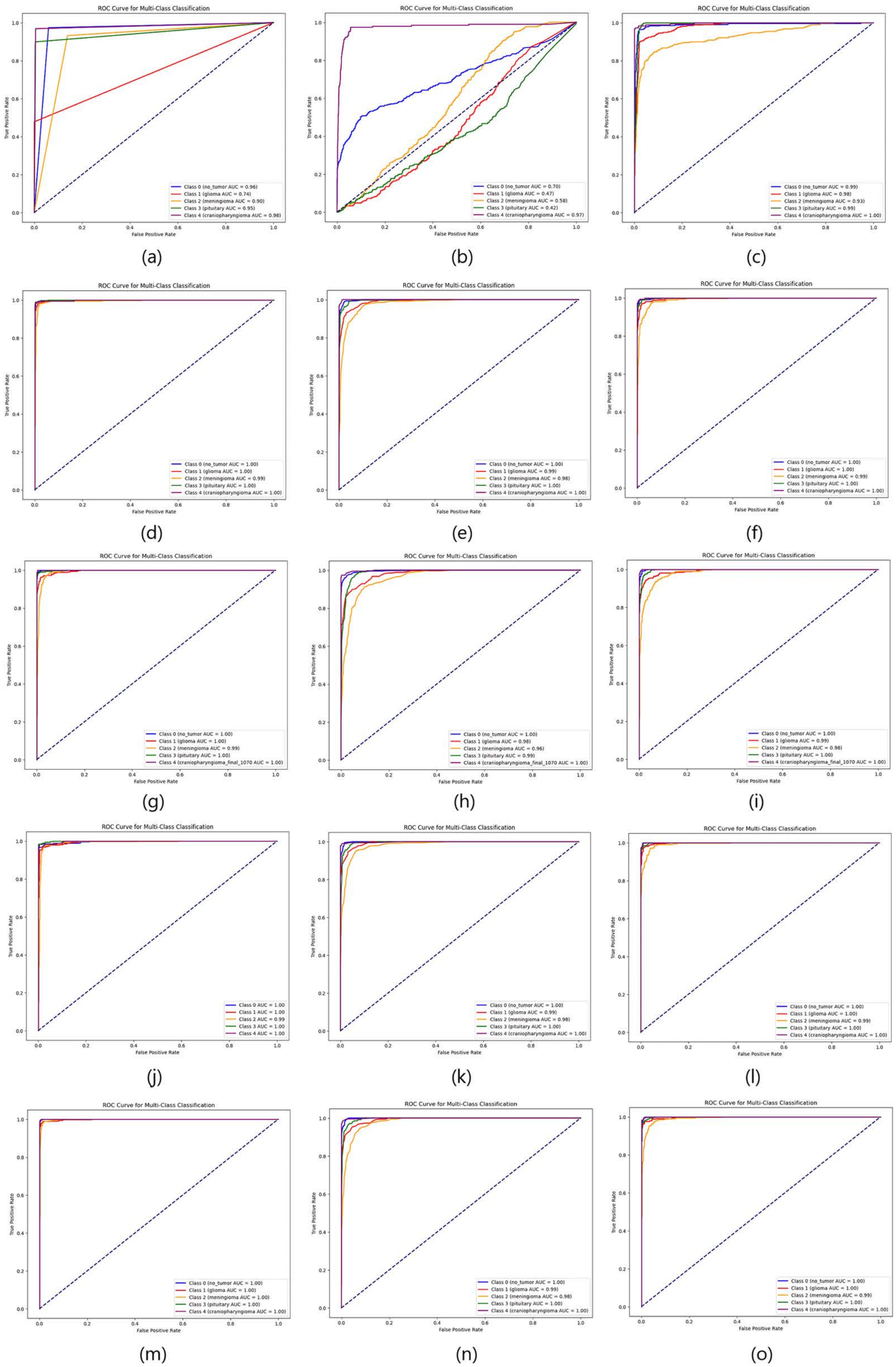


**Fig 9. Comparison of AUC curves on balanced dataset.** (a) CNN, (b) ResNet50, (c) EDL, (d) VGG16 + SVM, (e) VGG16 + RF, (f) VGG16 + XGB, (g) InceptionResnetV2 + SVM, (h) InceptionResnetV2 + RF, (i) InceptionResnetV2 + XGB, (j) DenseNet201 + SVM, (k) DenseNet201 + RF, (l) DenseNet201 + XGB, (m) DeepEFE + SVM, (n) DeepEFE + RF, (o) DeepEFE + XGB.

https://doi.org/10.1371/journal.pone.0310748.g009

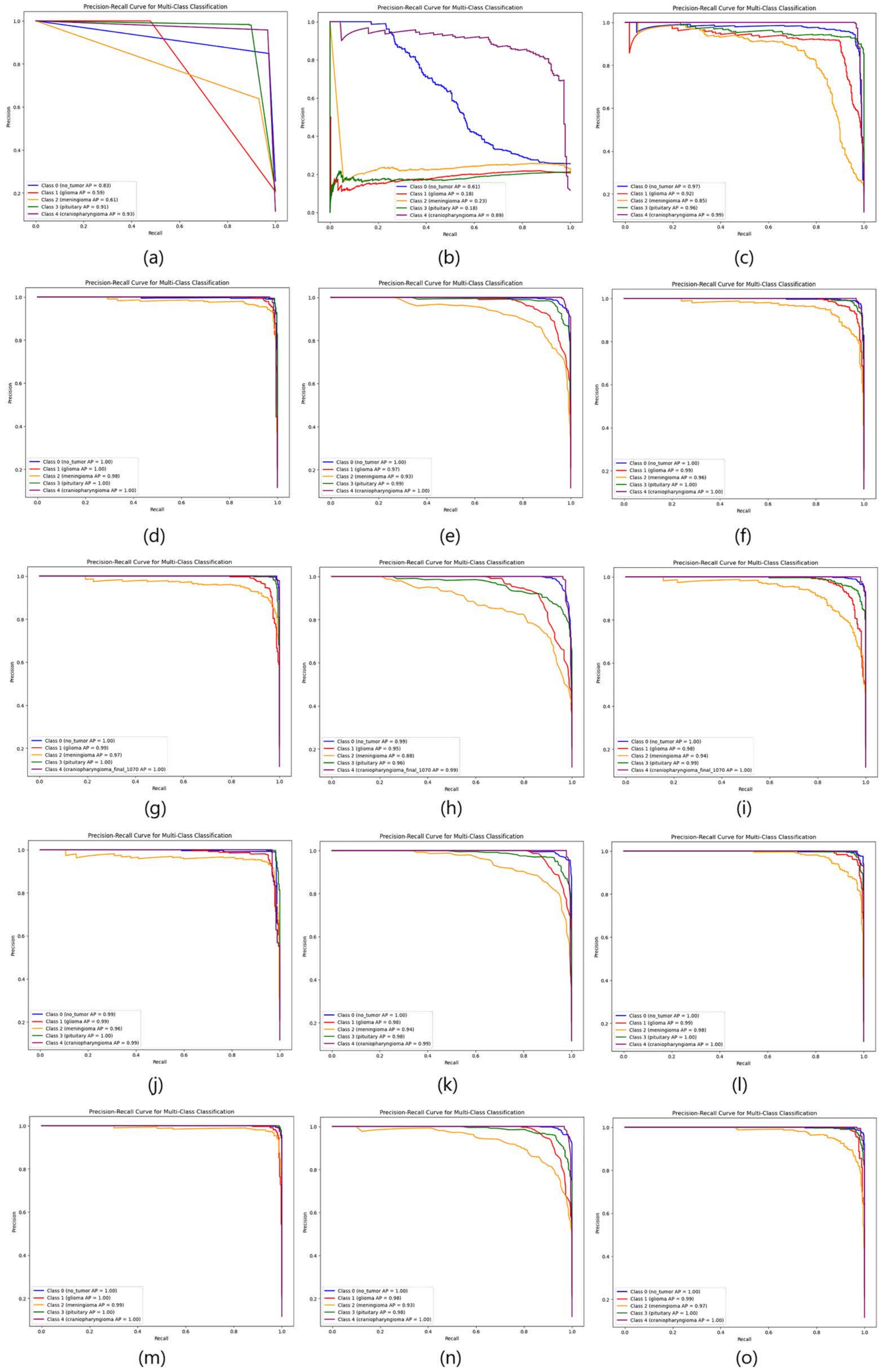


**Fig 10. Comparison of Precision-recall curves for balanced dataset.** (a) CNN, (b) ResNet50, (c) EDL, (d) VGG16 + SVM, (e) VGG16 + RF, (f) VGG16 + XGB, (g) InceptionResnetV2 + SVM, (h) InceptionResnetV2 + RF, (i) Inception-ResnetV2 + XGB, (j) DenseNet201 + SVM, (k) DenseNet201 + RF, (l) DenseNet201 + XGB, (m) DeepEFE + SVM, (n) DeepEFE + RF, (o) DeepEFE + XGB.

https://doi.org/10.1371/journal.pone.0310748.g010

still fall short compared to others. However, the minority class acted better than before even in these two model's outcomes. The higher these curve values are, the better and stable the models are performing.

Fig 11 presented below illustrates the generator and discriminator loss curves for the WGAN-GP. Notably, these curves depict a gradual convergence, underscoring the efficacy of our hybrid Dual-GAN mechanism in effectively generating synthetic samples to manage the imbalanced dataset. Real-ESRGAN used an improved VGG-style discriminator, a U-net discriminator with spectral normalization that is capable of addressing a much larger degradation space. This design outputs realness values for every pixel and provides a better per-pixel performance resulting in capturing more realistic information. This convergence signifies the remarkable progress made in our research and reinforces the applicability of our approach in the challenging domain of medical image analysis and highly skewed datasets.

## Validation results and performance evaluation

Cross-validation is a robust technique for model validation and evaluating the generalizability of a machine learning model. It provides insight into how the model is expected to perform on an independent dataset by partitioning the original dataset into a training set to train the model and a validation set to evaluate it. In this study, a 10-fold cross-validation is employed to assess the performance of the proposed DeepEFE model. The original dataset is split into ten (10) random partitions where the model is trained on nine (09) folds and the remaining fold is utilized for validation purpose.

Table 6 depicts the performance of the proposed model when combined with traditional ML classifiers. From the recorded results it is evident that DeepEFE demonstrates a stable and consistent performance across all the folds with average accuracy scores of 97.90%, 93.25%, and 95.64%, respectively. With significant Precision, Sensitivity, and F1-Scores the proposed model is proficient in accurate classification while maintaining minimal FPR and FNR rates listed in Table 7. These optimal outcomes infer that the proposed DeepEFE is a highly generalized, reliable, and efficient model, explicitly designed for classification tasks with its bias-free and accurate predictive quality.

During the cross-validation stages, the model accuracy for DeepEFE with SVM performed some fluctuations within a very limited range as depicted in Fig 12 which reflects the stability of the proposed combination. The highest accuracy achieved by the model in a fold is 98.52%. The precision-recall curve per fold for the proposed DeepEFE + SVM model is illustrated in Fig 13. And, the average PR curve for the model after a 10-fold CV is illustrated in the next figure (Fig 14). With no PR curve value lower than 0.99 it refers to the model's stable performance in classifying brain tumors. The next figure shows the confusion matrix of the model (Fig 15). With minimal misclassification, the confusion matrix reflects the tabulated results and resonates with the proposed model's superiority.

Similarly, the next few figures illustrate the performance of the DeepEFE model coupled with Random Forest after 10-fold cross-validation. During the cross-validation stages, 94.94% is the highest accuracy that could be achieved by this model as seen in Fig 16 and is comparatively lower than the preceding model. Moreover, the outcomes showed more oscillation during the folds. The precision-recall curve per fold for the DeepEFE + RF model is depicted in Fig 17. Although the curve possesses a significant AUC space, it is lower if compared with the model having the SVM classifier. One class is observed underperforming than the others in every fold. The same scenario prevailed through the average PR curve (Fig 18).

The DeepEFE + XGB stands somewhere between the previous two models regarding performance. It is evident from the accuracy results of the model over 10 folds illustration (Fig 19).

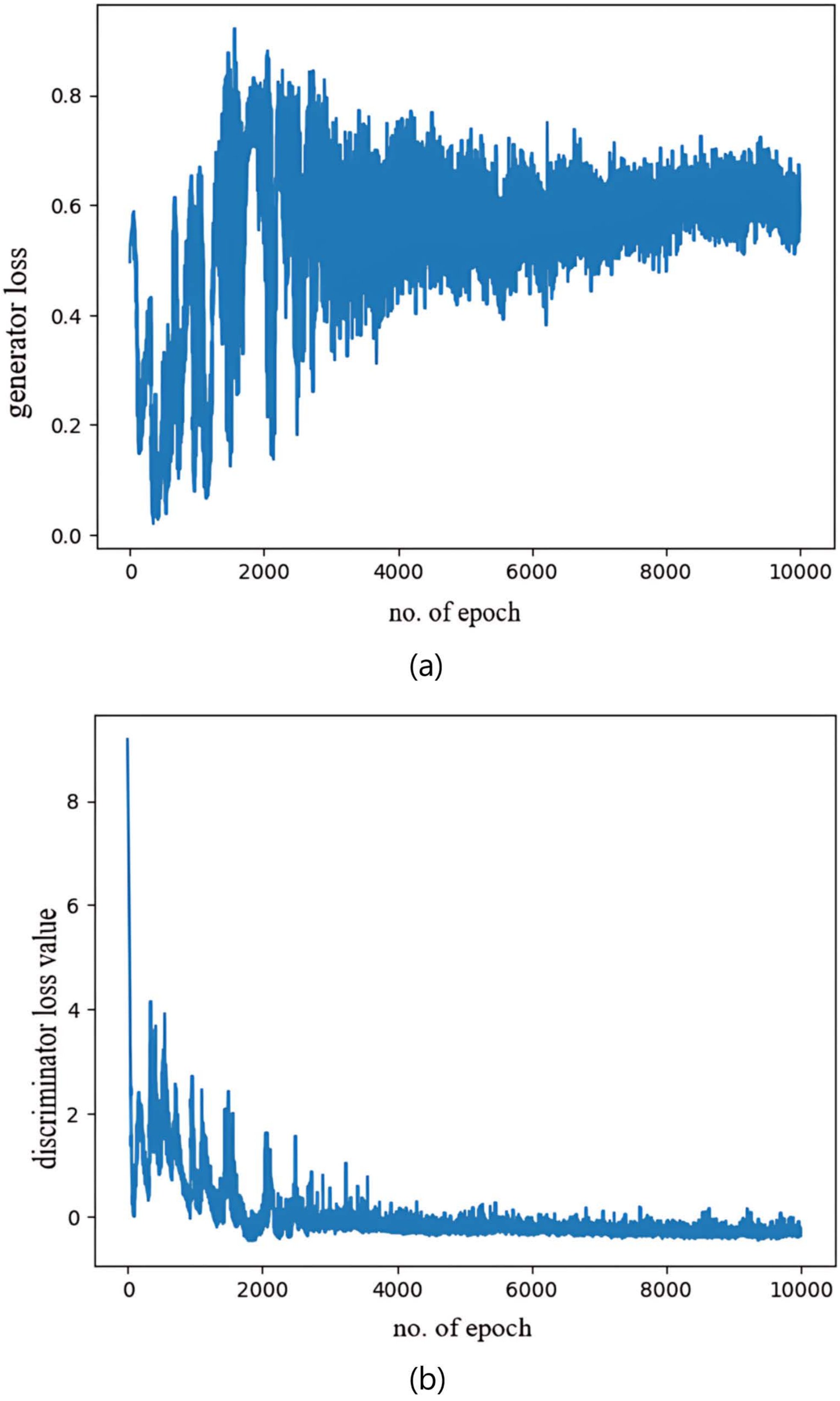


**Fig 11. Proposed model's generator and discriminator loss.** (a) The generator's loss shrinks into a stable range depicting the generator's ability to effectively produce real-like synthetic images. (b) The discriminator loss gradually reaches a low point portraying the discriminator's ability to distinguish real and fake images.

https://doi.org/10.1371/journal.pone.0310748.g011

**Table 6. Evaluation of Performance Using Cross-Validation.**

| Model | Fold | Accuracy | Precision | Sensitivity | F1-Score | Avg. Acc. (%) |
|---|---|---|---|---|---|---|
| DeepEFE + SVM | 1 | 98.27 | 98.30 | 98.27 | 98.28 | 97.9 |
| | 2 | 98.27 | 98.32 | 98.27 | 98.28 | |
| | 3 | 98.40 | 98.41 | 98.40 | 98.40 | |
| | 4 | 97.04 | 97.07 | 97.04 | 97.05 | |
| | 5 | 98.02 | 98.08 | 98.02 | 98.03 | |
| | 6 | 97.65 | 97.66 | 97.65 | 97.66 | |
| | 7 | 98.52 | 98.55 | 98.52 | 98.52 | |
| | 8 | 98.02 | 98.04 | 98.02 | 98.03 | |
| | 9 | 97.04 | 97.14 | 97.04 | 97.04 | |
| | 10 | 97.78 | 97.83 | 97.78 | 97.80 | |
| DeepEFE + RF | 1 | 94.45 | 94.73 | 94.45 | 94.50 | 93.25 |
| | 2 | 92.48 | 92.90 | 92.48 | 92.50 | |
| | 3 | 94.20 | 94.50 | 94.20 | 94.24 | |
| | 4 | 93.09 | 93.36 | 93.09 | 93.12 | |
| | 5 | 92.60 | 92.96 | 92.60 | 92.63 | |
| | 6 | 92.96 | 93.22 | 92.96 | 92.95 | |
| | 7 | 94.94 | 95.05 | 94.94 | 94.95 | |
| | 8 | 93.83 | 94.18 | 93.83 | 93.86 | |
| | 9 | 90.99 | 91.48 | 90.99 | 91.03 | |
| | 10 | 92.96 | 93.27 | 92.96 | 92.98 | |
| DeepEFE + XGB | 1 | 96.42 | 96.54 | 96.42 | 96.44 | 95.64 |
| | 2 | 95.07 | 95.20 | 95.07 | 95.08 | |
| | 3 | 96.18 | 96.22 | 96.18 | 96.17 | |
| | 4 | 95.31 | 95.38 | 95.31 | 95.31 | |
| | 5 | 95.80 | 95.90 | 95.80 | 95.80 | |
| | 6 | 95.06 | 95.15 | 95.06 | 95.06 | |
| | 7 | 96.67 | 96.73 | 96.67 | 96.68 | |
| | 8 | 95.93 | 96.03 | 95.93 | 95.95 | |
| | 9 | 94.57 | 94.93 | 94.57 | 94.59 | |
| | 10 | 95.43 | 95.54 | 95.43 | 95.44 | |

This table demonstrates the Accuracy, Precision, Recall, and F1-Score across all the folds of the cross-validation technique. The values signify that DeepEFE coupled with SVM, notably surpasses other models in all the performance metrics.

https://doi.org/10.1371/journal.pone.0310748.t006

Likewise, the PR curves show a balanced and positive classification efficiency both during individual folds Fig 20 and in average Fig 21. The proposed DeepEFE model has shown a stable and reliable performance, especially when it was coupled with the SVM classifier. The analysis of the model performances reverberates the proposed model's precision and generalizability in classification task.

## Explainabilty analysis

Explainability AI (XAI) is a vital aspect in the machine-learning research domain to explain the underlying mechanism of every model or functionality. Because, in a sensitive domain like the medical one, one of the major questions arises regarding the trustworthiness of the AI results and predictions. Therefore, these XAI techniques are helpful to bridge that trust gap. Gradient-Weighted Class-Activation Maps (Grad-CAM) is an XAI tool that is generally used in deep learning models to distinguish the input images' parts that contribute to the

**Table 7. Class-specific Cross Validation Performance Evaluation.**

| Model | Class | TPR | TNR | FPR | FNR | Specificity |
|---|---|---|---|---|---|---|
| DeepEFE + SVM | no_tumor | 0.99 | 1.00 | 0.00 | 0.01 | 0.99 |
| | glioma | 0.96 | 1.00 | 0.00 | 0.04 | 0.99 |
| | meningioma | 0.97 | 0.99 | 0.01 | 0.03 | 0.98 |
| | pituitary | 0.99 | 1.00 | 0.00 | 0.01 | 1.00 |
| | craniopharyngioma | 0.97 | 1.00 | 0.00 | 0.03 | 1.00 |
| DeepEFE + RF | no_tumor | 0.97 | 0.99 | 0.01 | 0.03 | 0.99 |
| | glioma | 0.88 | 0.99 | 0.01 | 0.12 | 0.99 |
| | meningioma | 0.88 | 0.96 | 0.04 | 0.12 | 0.95 |
| | pituitary | 0.98 | 0.96 | 0.04 | 0.02 | 0.95 |
| | craniopharyngioma | 0.94 | 1.00 | 0.00 | 0.06 | 1.00 |
| DeepEFE + XGB | no_tumor | 0.98 | 0.99 | 0.01 | 0.01 | 0.99 |
| | glioma | 0.92 | 1.00 | 0.00 | 0.08 | 0.99 |
| | meningioma | 0.93 | 0.97 | 0.03 | 0.07 | 0.97 |
| | pituitary | 0.98 | 0.98 | 0.02 | 0.02 | 0.98 |
| | craniopharyngioma | 0.96 | 1.00 | 0.00 | 0.04 | 1.00 |

The table represents the performance of each model against each class after performing cross-validation. Noteworthily, the proposed DeepEFE combined with SVM exhibits superior performance securing the highest sensitivity rates with lesser error ratio.

https://doi.org/10.1371/journal.pone.0310748.t007

prediction of a particular class [47]. Therefore, we have utilized this technique to produce heatmaps overlaying the input images, with red regions denoting high importance and gradually bluish regions indicating progressively decreasing importance. It elucidates the model's decision boundaries and identifies potential areas of misinterpretation.

The associated Grad-CAM function calculates the importance map by deriving the reduction layer output's derivative for a designated class with respect to a convolutional feature map, intelligently selecting suitable layers for the task in classification scenarios. Fig 22 represents the Grad-CAM analysis on one image from each of the classes that are randomly chosen by the model. It is observed that Grad-CAM accurately highlights the critical area of interest in red, aligning with our research outcomes. From the visualization, we can locate the tumor sites precisely and identify the tumor to distinguish it from the tumor-negative class. However, to some extent, we have encountered cases where Grad-CAM has assigned a mixed color palette or assigned red color beyond the critical regions. This suggests the potential challenges in the model's accurate prediction outcome. Therefore, while Grad-CAM provides valuable insights into the model's attention mechanism, such scenarios strongly suggest that there is still scope to improve the existing XAI tools to convert the black-box artificial intelligence algorithms into glass-box models.

## Comparative analysis and discussion

Table 8 provides an in-depth analysis of the models employed and the corresponding results, both in relevant studies found during the literature review and in the proposed methodology. The table highlights an important finding: previous research has mainly focused on using deep features for brain tumor classification, but has only achieved moderately satisfactory accuracies. This underscores a substantial opportunity for further progress and innovation within this domain. Furthermore, it is worth noting that previous studies on brain tumor classification have not adequately tackled the problem of imbalanced datasets. The majority of these studies have primarily focused on binary classification tasks neglecting the heterogeneity of the input data. This study, in contrast, introduces a highly effective pipeline to address the

**Table 8. Comparison with Baseline Studies.**

| Author | Year | Dataset | Model | Accuracy |
|---|---|---|---|---|
| Afshar et al. | 2020 | Public Dataset (3 classes) | Boosted Capsule Networks | 92.45% |
| Ali et al. | 2023 | Public Dataset (4 classes) | WBM-DLNet with SVM | 95.70% |
| Sujatha & Rao | 2023 | Public Dataset | DenseNet201 | 88.00% |
| Bitto et al. | 2023 | Private Dataset | ResNet-50 | 96.76% |
| Najeeb et al. | 2022 | Public Dataset | Deep Features | 90.20% |
| Toğą car et al. | 2020 | Public Dataset (2 classes) | CNN with attention modules and hypercolumn technique | 96.05% |
| Ramamoorthy et al. | 2022 | Private Dataset (2 classes) | Adaptive Histogram Contrast Normalization with Learning-based Neural Quantization | 93.00% |
| Shimanto et al. [48] | 2023 | Public Dataset (4 classes) | Deep Features with SVM | 97.14% |
| Khan et al. [49] | 2022 | Public Dataset (4 classes) | 23-layers CNN model | 97.80% |
| Díaz-Pernas et al. [50] | 2021 | Public Dataset (4 classes) | CNN | 97.00% |
| Dipu et al. [51] | 2021 | Public Dataset (2 classes) | FastAi | 95.78% |
| Saxena et al. [52] | 2020 | Public Dataset (2 classes) | ResNet50 | 95.00% |
| Gudigar et al. [53] | 2019 | Public Dataset (2 classes) | Particle Swarm Optimization with SVM | 97.40% |
| Hemanth et al. [54] | 2019 | Public Dataset (4 classes) | DCNN | 95.00% |
| **This Study** | 2024 | Combined Dataset from 3 Publicly Available Ones (5 classes) | Deep Ensemble Feature Extraction combined with SVM | **98.15%** |

The table compares the existing approaches with the proposed model and highlights the supremacy of our proposed DeepEFE model. The proposed study addresses a comparatively greater number of classes.

https://doi.org/10.1371/journal.pone.0310748.t008

challenge of imbalanced diversified datasets, while also significantly improving the accuracy of brain tumor classification. By addressing the imbalanced dataset challenge and prioritizing multi-class classification, the study seeks to elevate the accuracy and robustness of brain tumor classification algorithms.

Fig 23 further demonstrates the outcomes derived from the hybrid pipeline implemented. The figure above clearly demonstrates that the combination of DeepEFE and SVM outperforms previous studies, with the highest average accuracy achieved. These promising results suggest that the integration of DeepEFE with SVM has the potential to improve accuracy in multi-class classification tasks significantly.

## Conclusion and future work

Detecting a brain tumor poses a significant challenge due to the intricate nature of the brain's structure. This study has navigated the complex landscape of brain tumor classification through the amalgamation of diverse machine learning and deep learning techniques. Dealing with imbalanced medical datasets presents a significant challenge in ensuring proper healthcare as it frequently leads to biased behavior favoring the majority classes. This research aims to address the critical issue of brain tumors by presenting a comprehensive and reliable pipeline for early-stage classification of brain tumors. The significance of this pipeline cannot be exaggerated, as it plays a crucial role in addressing the growing prevalence of brain tumor-related diseases worldwide.

The proposed pipeline incorporates a hybrid dual-GAN mechanism producing realistic synthetic images from the original image data, effectively. The hybrid dual-GAN mechanism utilizes both WGAN-GP and Real-ESRGAN to improve the quality of these newly created image data. This approach aims to address the imbalance in the MRI dataset and produce more realistic artificial images for improved training and analysis. Furthermore, the pipeline incorporates advanced ensemble feature extraction techniques to extract meaningful features

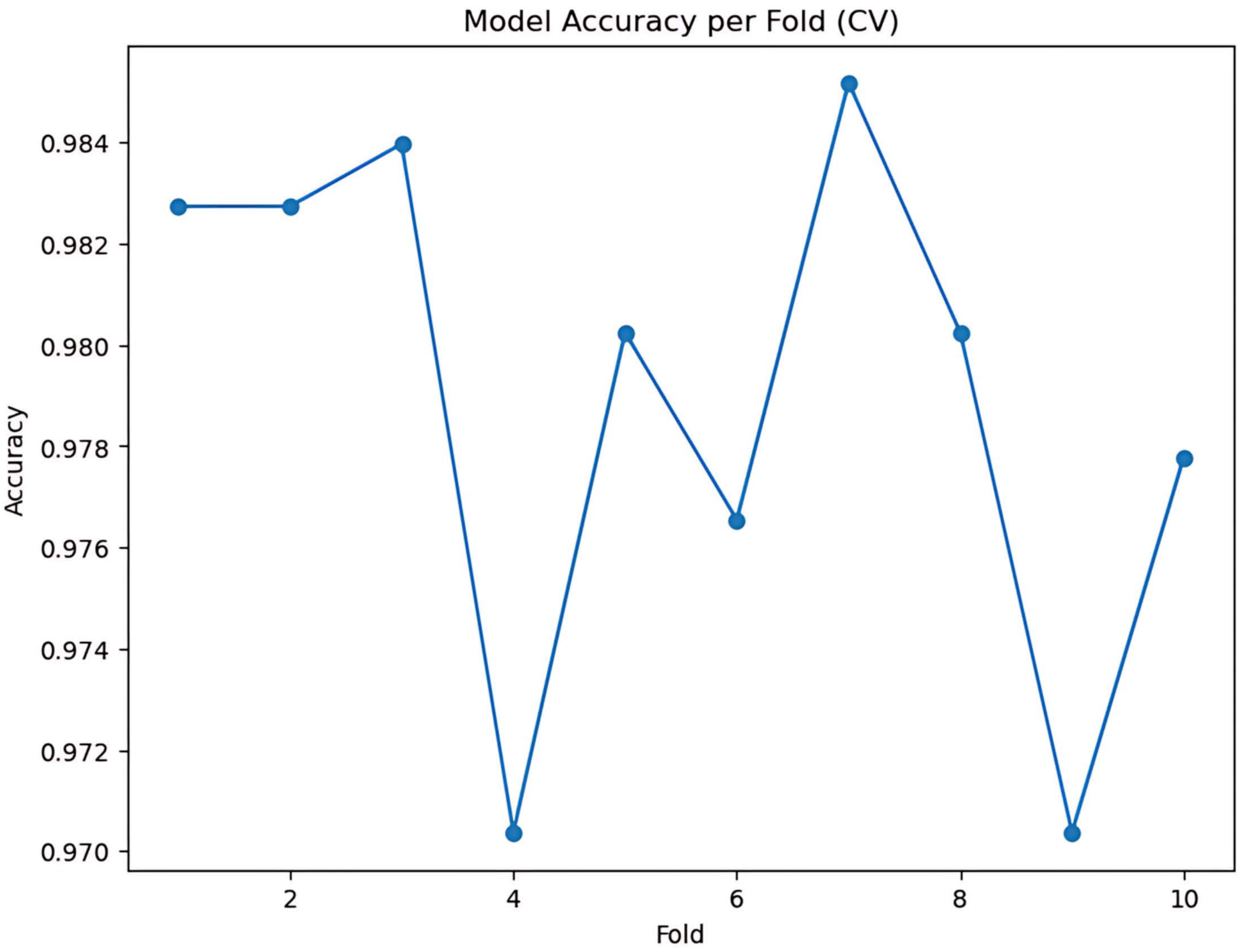


**Fig 12. (DeepEFE + SVM) model performance over 10-fold cross-validation.**

https://doi.org/10.1371/journal.pone.0310748.g012

from medical imaging data, allowing for accurate and early detection of potential brain tumors. By leveraging advanced ML and deep learning algorithms, the model can classify brain tumor cases with high precision, paving the way for timely intervention and improved patient outcomes.

The proposed Deep Ensemble Feature Extraction (DeepEFE) model, when combined with SVM, attained a remarkable accuracy of 98.15% surpassing other cutting-edge deep learning models. Moreover, the results obtained from 10-fold cross-validation demonstrate the robustness and generalizability of the proposed model. The model's consistent performance across all folds underscores its reliability, implying that it will excel with unseen data. This noteworthy performance demonstrates the effectiveness of combining feature extraction techniques with support vector machines for classification tasks. Furthermore, it showcases the potential of the proposed DeepEFE model in achieving high-performance results in multi-class classification. This study incorporates Grad-CAM to better understand the significantly contributing

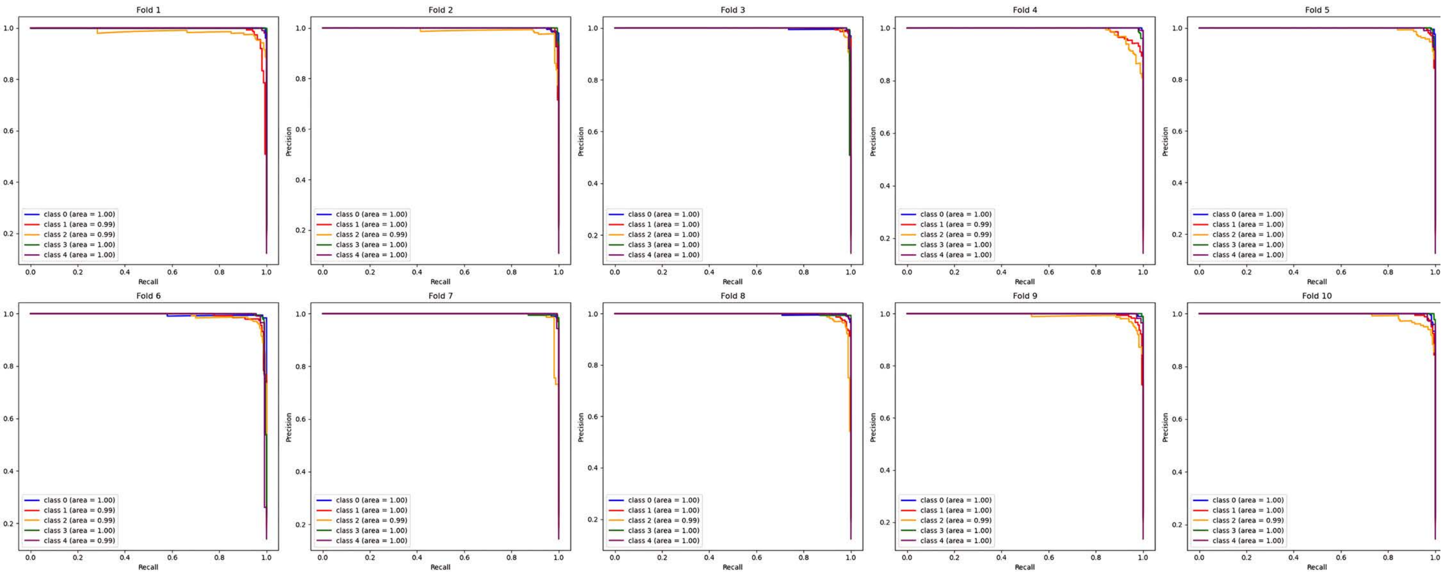


**Fig 13. Precision-recall curve at each fold for (DeepEFE + SVM) model.**

https://doi.org/10.1371/journal.pone.0310748.g013

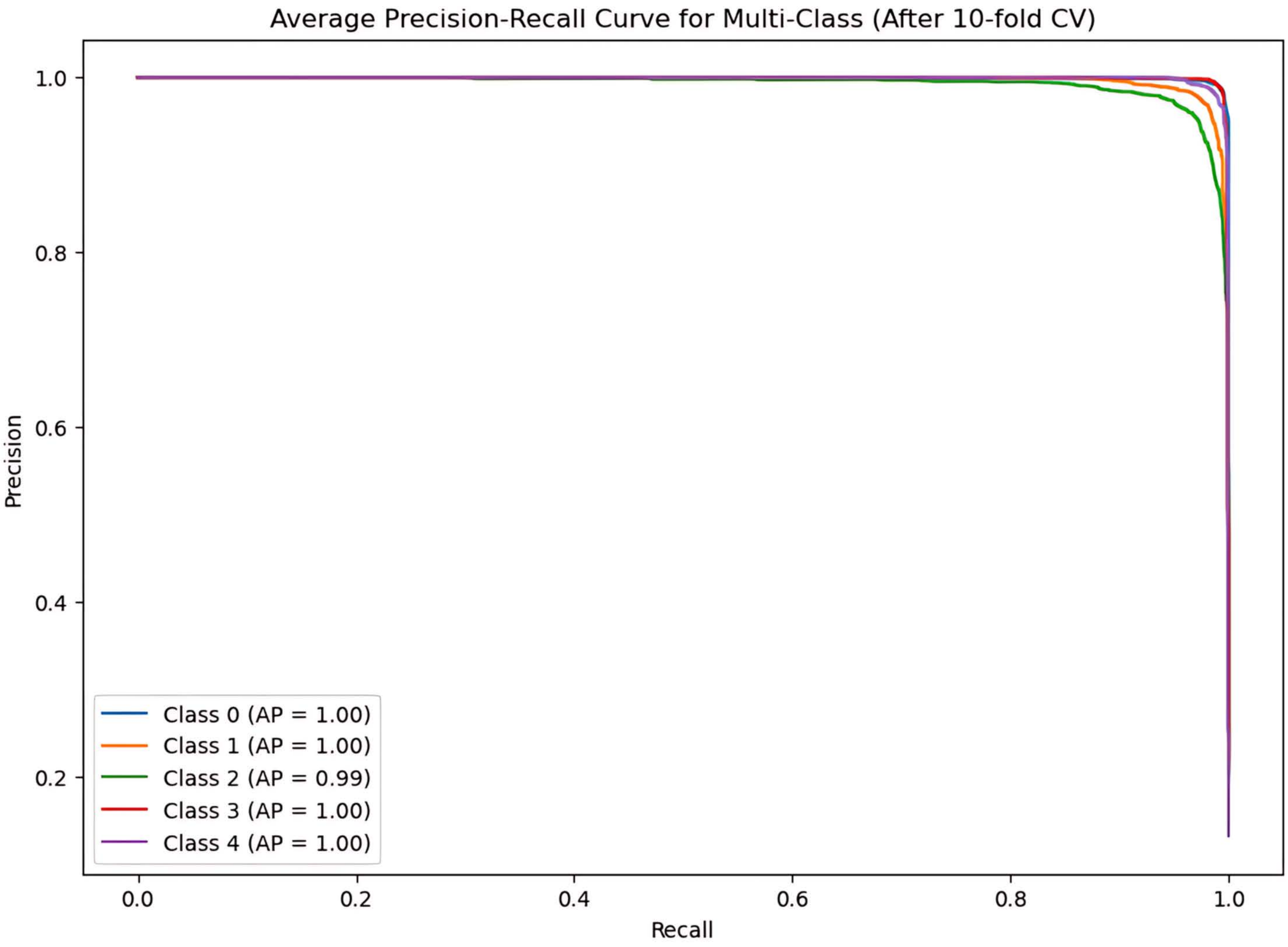


**Fig 14. The average Precision-recall curve for (DeepEFE + SVM) model after 10-Fold CV.**

https://doi.org/10.1371/journal.pone.0310748.g014

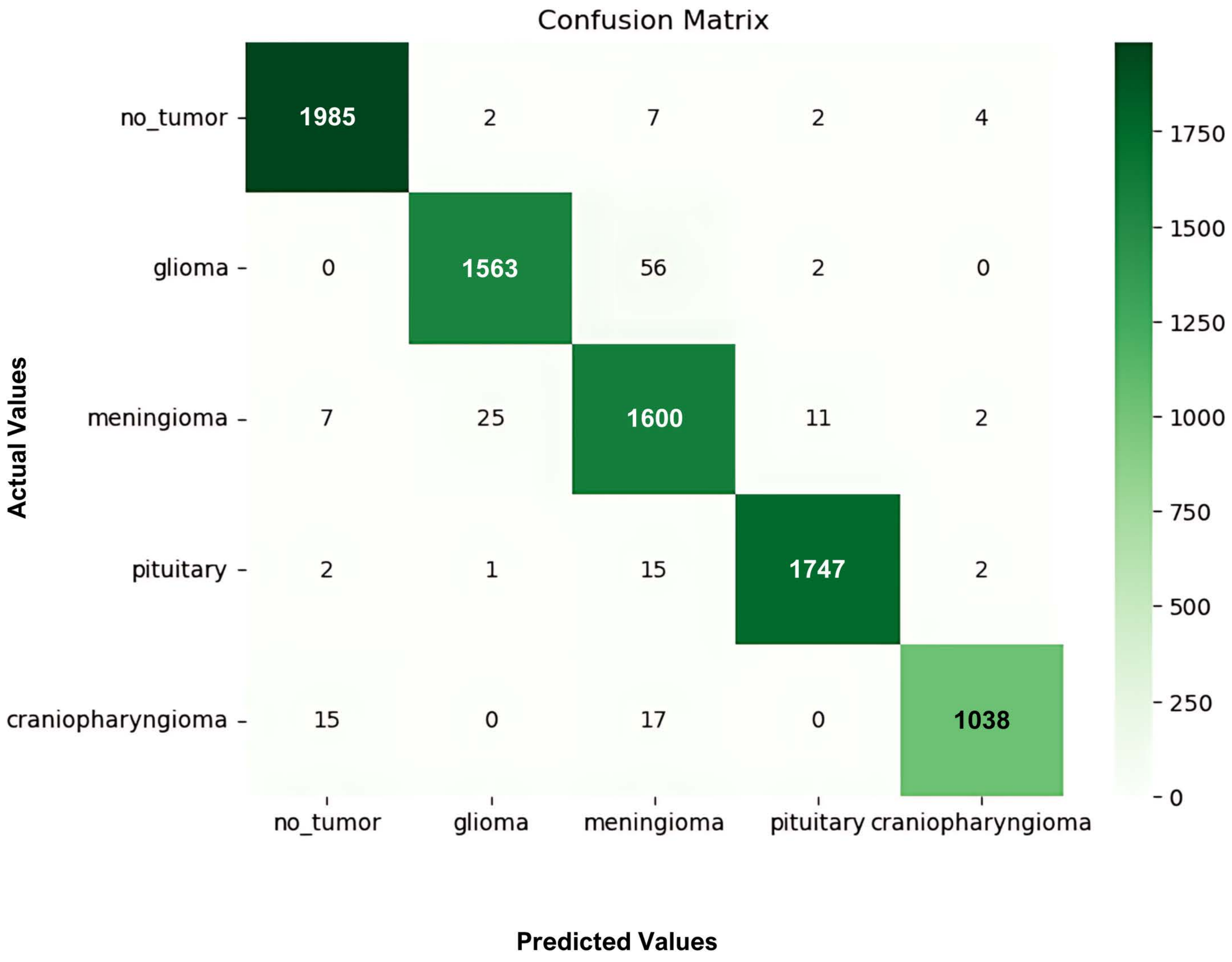


**Fig 15. Confusion matrix for the proposed (DeepEFE + SVM) after 10-fold CV.**

https://doi.org/10.1371/journal.pone.0310748.g015

parts of the input images. This makes the proposed mechanism more reliable by turning black-box models into easier-to-understand glass-box techniques. However, these XAI tools need further development for more accurate precision since they marked a few non-contributing regions as well which can lead to misinterpretation of the model. The current paper contributes to the field of medical diagnostics as the insights provided by Grad-CAM enhance the transparency and trustworthiness of the model's predictions. It lays the groundwork for further advancements in brain tumor classification integrating distributed ML approaches on more complex datasets.

We will prioritize the emphasis on ensuring user privacy and data confidentiality in our future work, as these concerns are of supreme importance in this 21$^{st}$ century, especially when working with medical data. To achieve this, we aim to implement robust encryption protocols and integrate distributed ML approaches to our proposed pipeline. This approach not only minimizes the risk of potential data breaches but also allows for a more collaborative and secure healthcare environment by ensuring model training across decentralized devices.

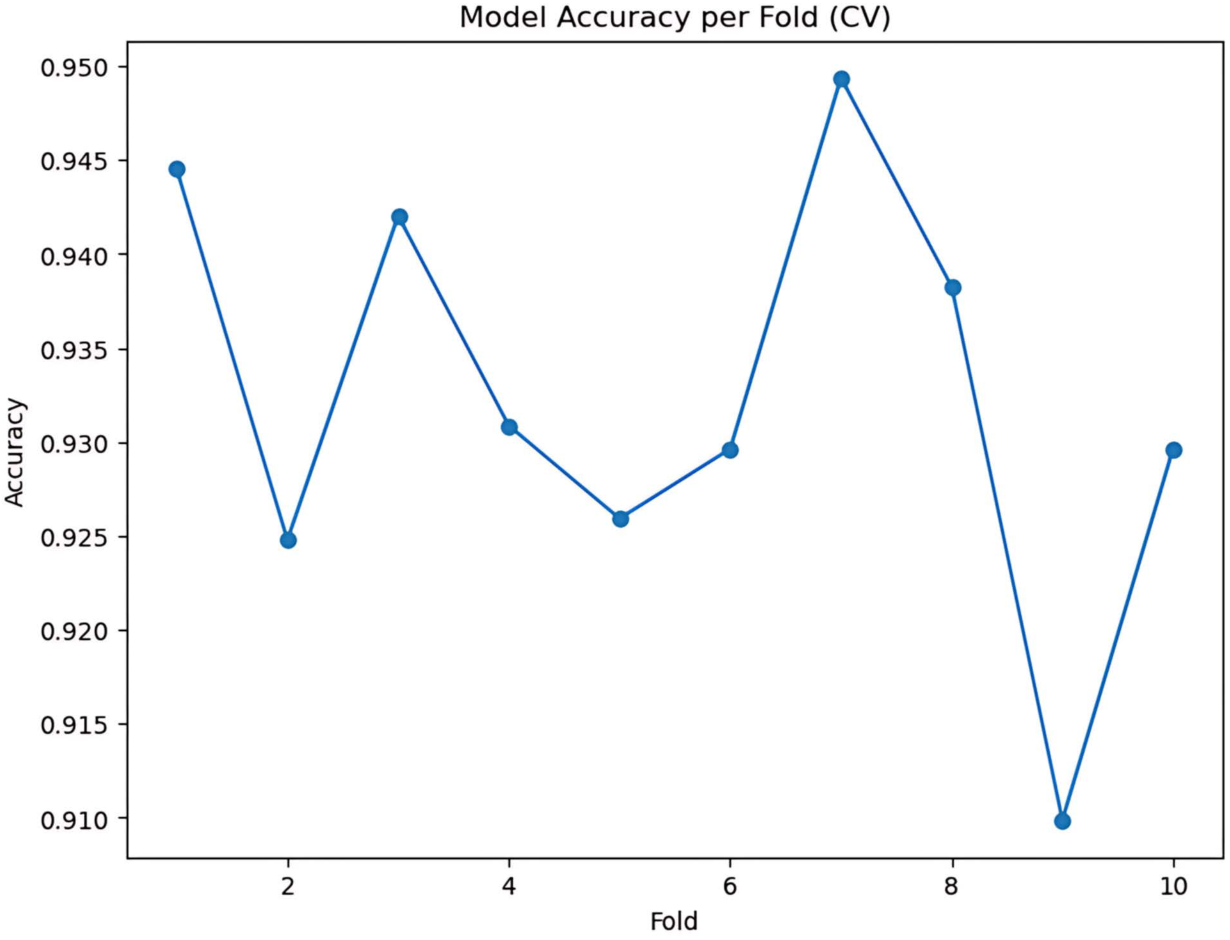


**Fig 16. (DeepEFE + RF) model performance over 10-Fold CV.**

https://doi.org/10.1371/journal.pone.0310748.g016

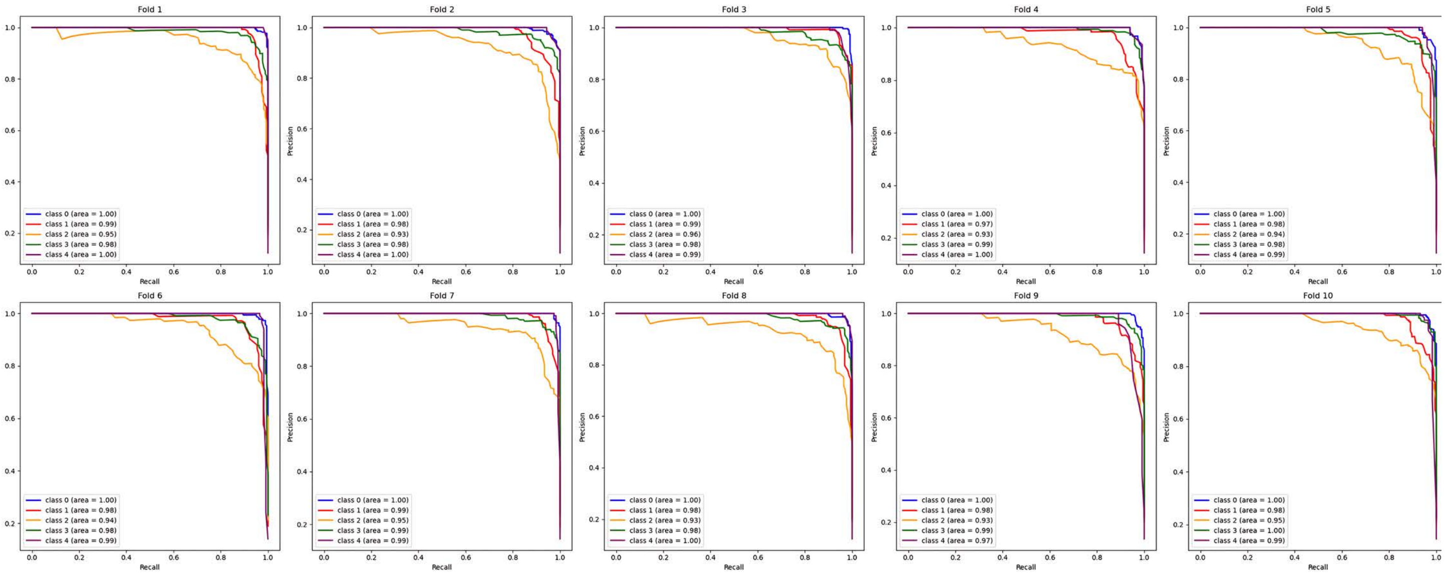


**Fig 17. Precision-recall curve at each fold for (DeepEFE + RF) model.**

https://doi.org/10.1371/journal.pone.0310748.g017

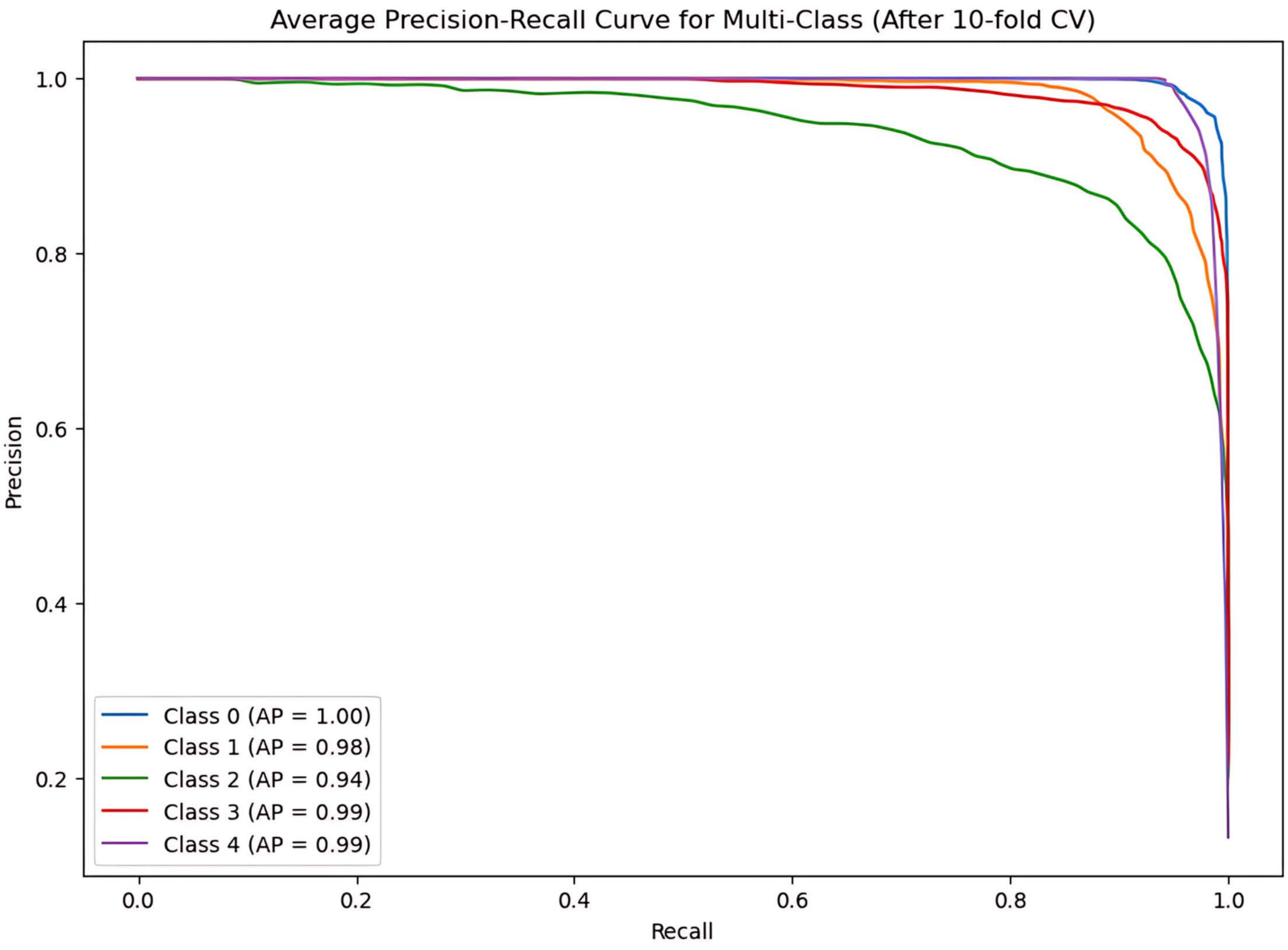


**Fig 18. The average precision-recall curve for (DeepEFE + RF) model after 10-Fold CV.**

https://doi.org/10.1371/journal.pone.0310748.g018

Additionally, we plan to analyze gene expression datasets to determine the efficacy of our proposed pipeline when trained on such datasets. We believe this integration will help experts gain valuable insights into how biological structures influence brain tumors ultimately leading to the development of more personalized treatment strategies based on individual gene expression patterns.

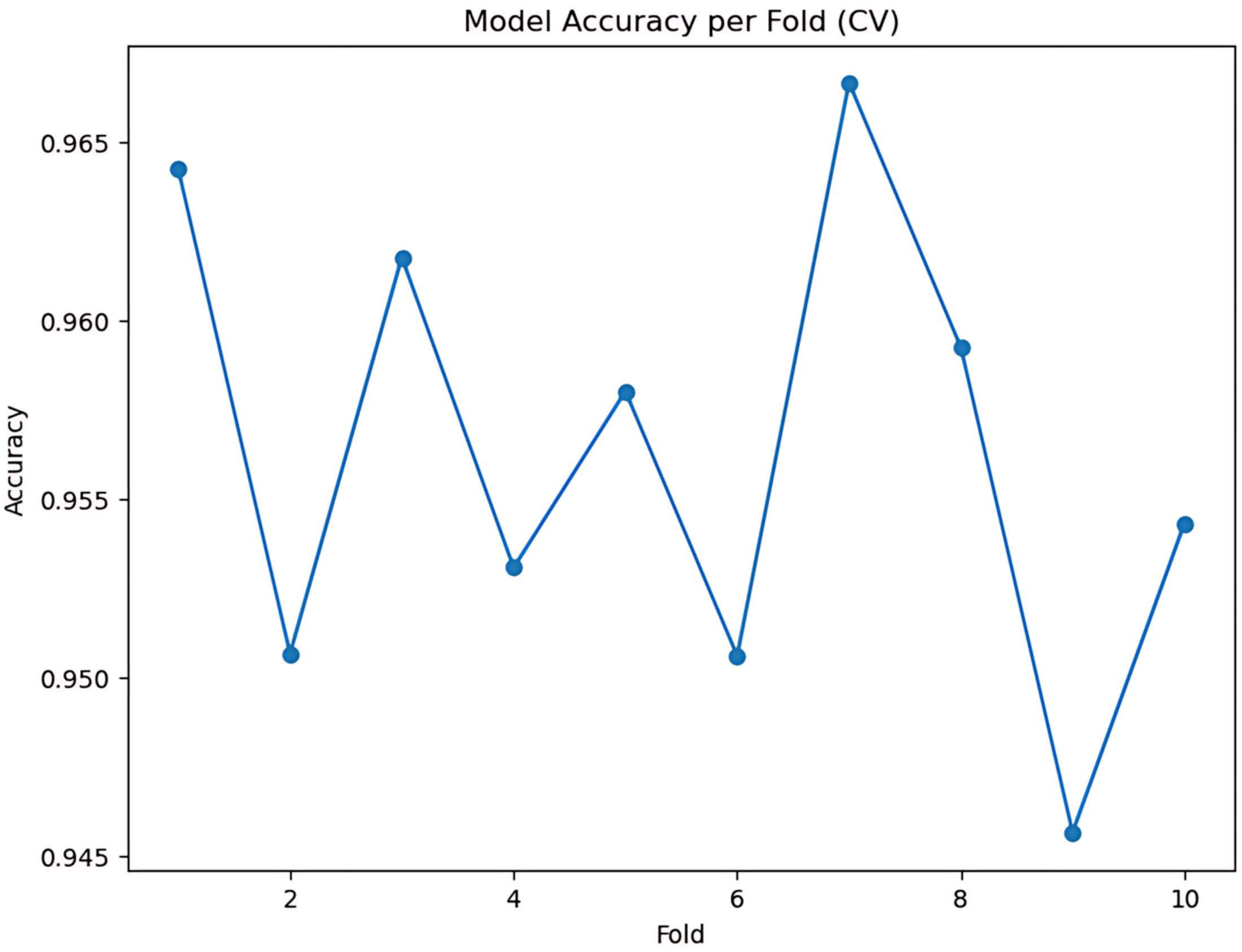


**Fig 19. (DeepEFE + XGB) model performance over 10-Fold CV.**

https://doi.org/10.1371/journal.pone.0310748.g019

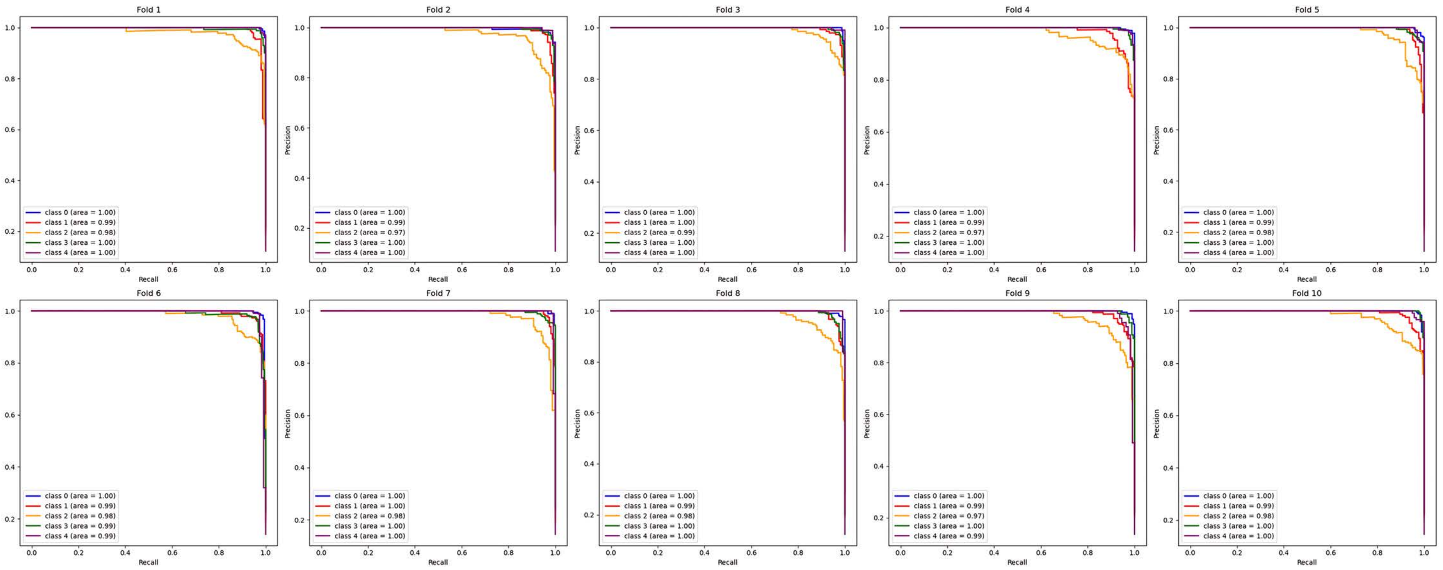


**Fig 20. Precision-recall curve at each fold for (DeepEFE + XGB) model.**

https://doi.org/10.1371/journal.pone.0310748.g020

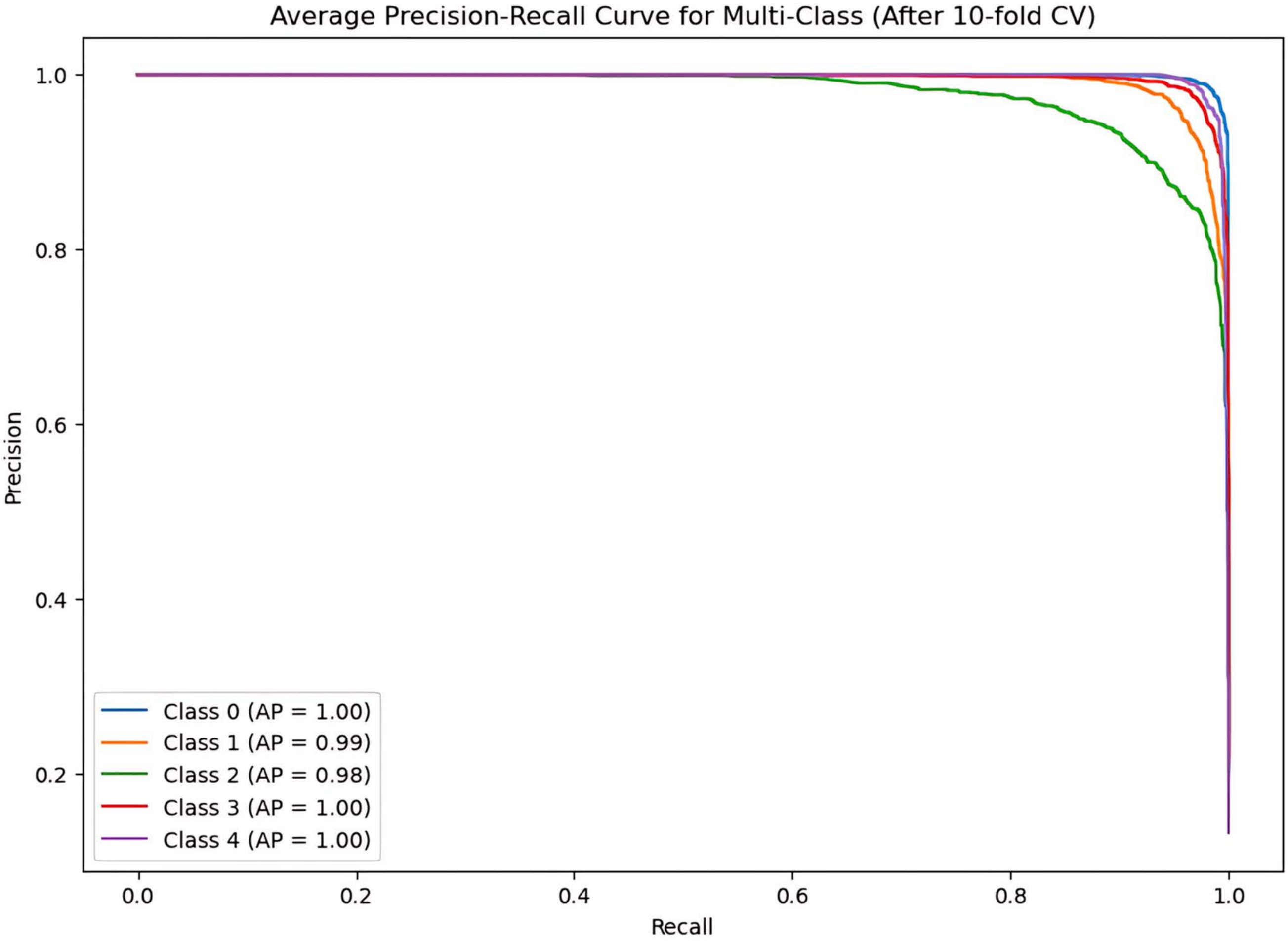


**Fig 21. The average Precision-recall curve for (DeepEFE + XGB) model after 10-fold CV.**

https://doi.org/10.1371/journal.pone.0310748.g021

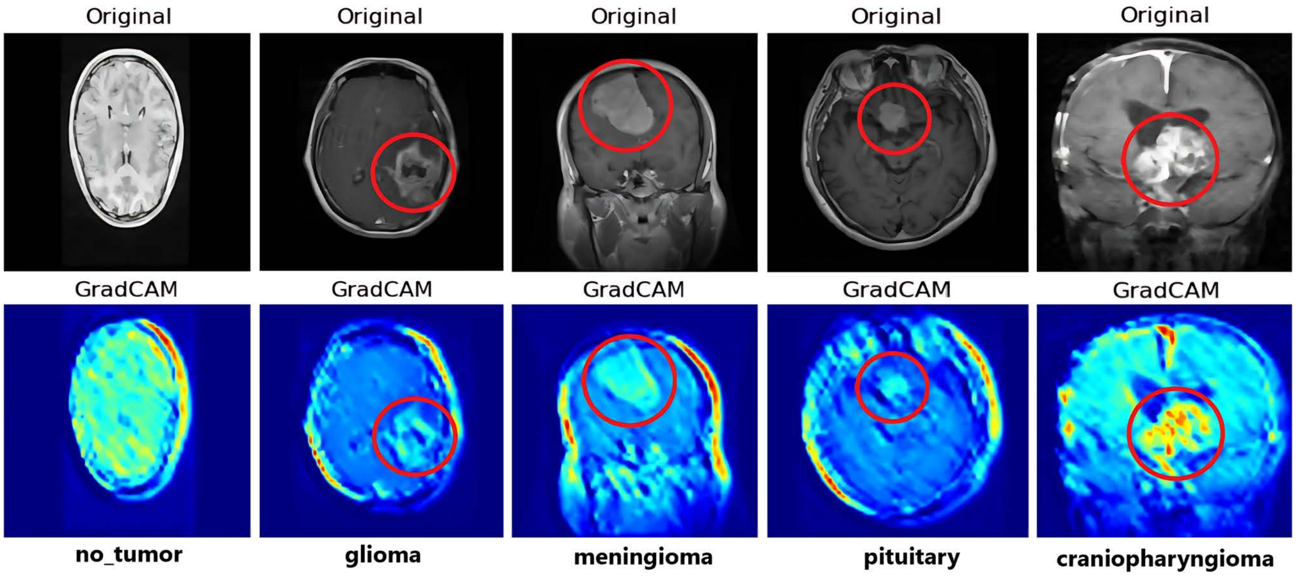


**Fig 22. Visualization with Grad-CAM for model explainability: Red regions correspond to high scores for the class and blue corresponds to low evidence for the class.**

https://doi.org/10.1371/journal.pone.0310748.g022

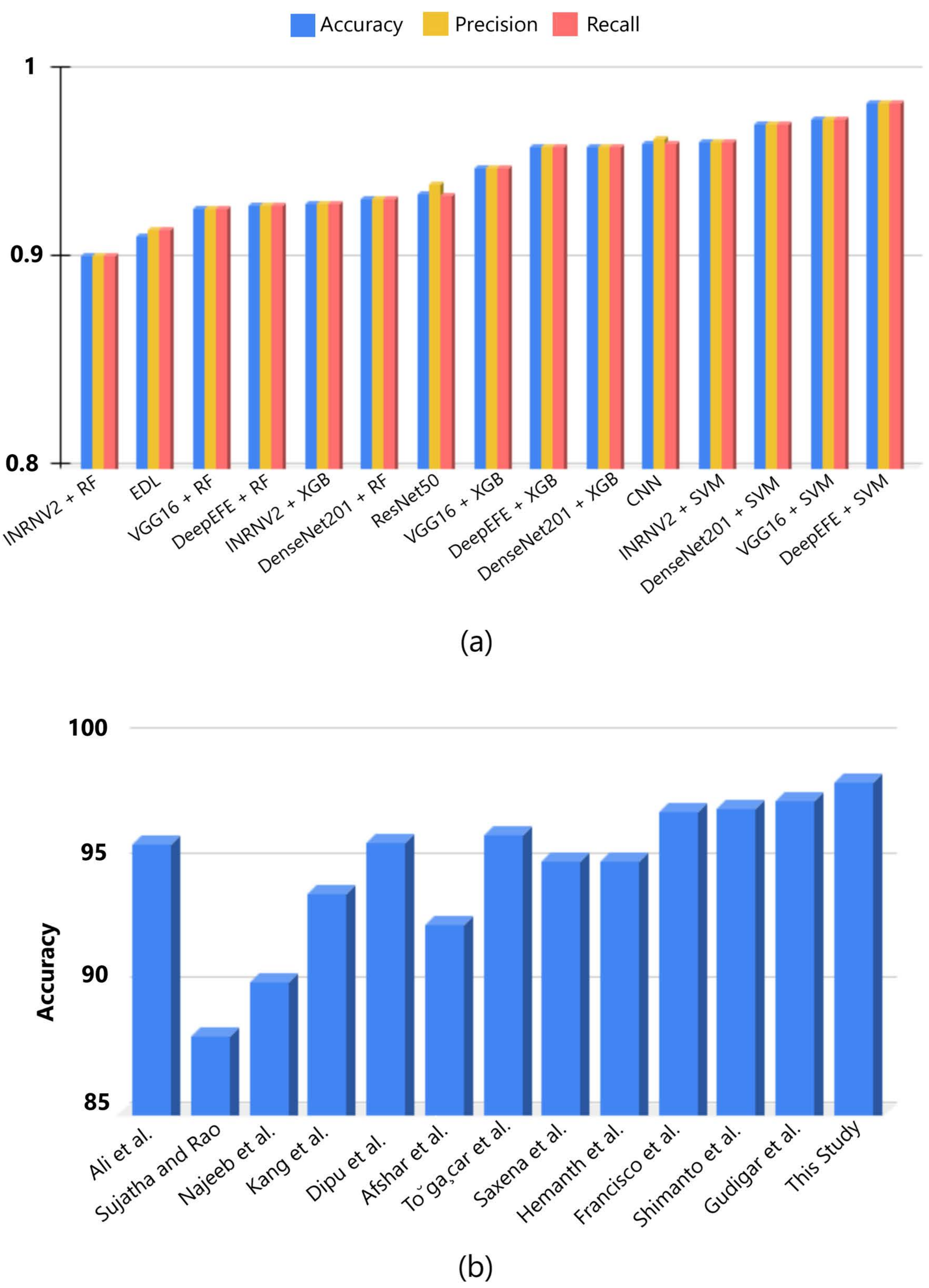


**Fig 23. Comparison with the baseline studies.** (a) The image illustrates the analyzed model's performance comparison. (b) The image compares the proposed model with other novel studies on brain tumor classification.

https://doi.org/10.1371/journal.pone.0310748.g023

## Author contributions

**Conceptualization:** Priyanka Roy.

**Data curation:** Priyanka Roy, Fahim Mohammad Sadique Srijon.

**Formal analysis:** Priyanka Roy, Fahim Mohammad Sadique Srijon.

**Investigation:** Priyanka Roy, Fahim Mohammad Sadique Srijon.

**Methodology:** Priyanka Roy.

**Supervision:** Pankaj Bhowmik.

**Validation:** Pankaj Bhowmik.

**Visualization:** Priyanka Roy, Fahim Mohammad Sadique Srijon.

**Writing – original draft:** Priyanka Roy, Fahim Mohammad Sadique Srijon.

**Writing – review & editing:** Pankaj Bhowmik.

## References

1. Mohan P., Veerappampalayam Easwaramoorthy S., Subramani N., Subramanian M., Meckanzi S. Handcrafted Deep-Feature-Based Brain Tumor Detection and Classification Using MRI Images. Electronics, vol. 11, no. 24, article number 4178, December 14, 2022. doi: 10.3390/electronics11244178
2. Brain tumor—Symptoms and causes—Mayo Clinic. Year: 2023. Accessed on 2023-10-26. Available online: https://www.mayoclinic.org/diseases-conditions/brain-tumor/symptoms-causes/.
3. Brain Tumor—Statistics. Year: 2023. Available online: https://www.cancer.net/cancer-types/brain-tumor/statistics.
4. Cancer of the Brain and Other Nervous System—Cancer Stat Facts. Accessed on 2024-03-12. Available at: https://seer.cancer.gov/statfacts/html/brain.html.
5. Brain Tumor—Statistics. Accessed on 2024-03-12. Available at: https://www.cancer.net/cancer-types/brain-tumor/statistics.
6. Anitha V. and Murugavalli S. Brain tumour classification using twotier classifier with adaptive segmentation technique. IET Computer Vision, vol. 10, no. 1, pp. 9–17, February 2016. doi: 10.1049/iet-cvi.2014.0193
7. Afshar, P. and Plataniotis, K. N. and Mohammadi, A. BoostCaps: A Boosted Capsule Network for Brain Tumor Classification. 2020 42nd Annual International Conference of the IEEE Engineering in Medicine & Biology Society (EMBC). 2020 July.
8. Cheng, Jun. Brain Tumor Dataset. figshare, 2017. Available online: https://doi.org/10.6084/m9.figshare.1512427.v5.
9. Shahzadi, I. and Tang, T. B. and Meriadeau, F. and Quyyum, A. CNN-LSTM: Cascaded Framework For Brain Tumour Classification. 2018 IEEE-EMBS Conference on Biomedical Engineering and Sciences (IECBES), December 2018.
10. The Multimodal Brain Tumor Image Segmentation Benchmark (BRATS). Available online: https://doi.org/10.1109/TMI.2014.2377694, October 1, 2015.
11. Ali Muhammad Umair, Hussain Shaik Javeed, Zafar Amad, Bhutta Muhammad Raheel, Lee Seung Won. WBM-DLNets: Wrapper-Based Metaheuristic Deep Learning Networks Feature Optimization for Enhancing Brain Tumor Detection. Bioengineering, vol. 10, no. 4, article number 475, 2023. Available online: https://www.mdpi.com/2306-5354/10/4/475. doi: 10.3390/bioengineering10040475 PMID: 37106662
12. Chakrabarty, N., Kanchan, S. Brain Tumor Classification (MRI). Available online: https://www.kaggle.com/datasets/sartajbhuvaji/brain-tumor-classification-mri?select=Training. Accessed on 17 March 2022.
13. Sujatha, K. and Rao, B. S. Densenet201: A Customized DNN model for Multi-Class Classification and Detection of Tumors Based on Brain MRI Images. 2023 Fifth International Conference on Electrical, Computer and Communication Technologies (ICECCT), February 22, 2023.
14. Bitto A. K., Bijoy M. H. I., Yesmin S., Mahmud I., Mia M. J., and Biplob K. B. B. Tumor-Net: convolutional neural network modeling for classifying brain tumors from MRI images. In: International Journal of Advances in Intelligent Informatics, volume 9, number 2, page 148. April 7, 2023. https://doi.org/10.26555/ijain.v9i2.872

15. Pilaoon, P., Maneerat, N., Nakthewan, A., Varakulsiripunth, R., Hamamoto, K. Brain Tumor Classification using Pretrained Deep Convolutional Neural Network. 2023 9th International Conference on Engineering, Applied Sciences, and Technology (ICEAST), June 1, 2023.
16. Mahamed Najeeb, R. S., Abdul Majjed Dahl, I. O. Brain Tumor Segmentation Utilizing Generative Adversarial, Resnet And Unet Deep Learning. 2022 8th International Conference on Contemporary Information Technology and Mathematics (ICCITM), August 31, 2022.
17. Toğaçar M., Ergen B., Cömert Z. BrainMRNet: Brain tumor detection using magnetic resonance images with a novel convolutional neural network model. Medical Hypotheses, vol. 134, p. 109531, January 2020. doi: 10.1016/j.mehy.2019.109531 PMID: 31877442
18. Shree N. Varuna, Kumar T. N. R. Identification and classification of brain tumor MRI images with feature extraction using DWT and probabilistic neural network. Brain Informatics, vol. 5, no. 1, pp. 23–30, January 8, 2018. doi: 10.1007/s40708-017-0075-5
19. Gumaei A., Hassan M. M., Hassan M. R., Alelaiwi A., Fortino G. A Hybrid Feature Extraction Method With Regularized Extreme Learning Machine for Brain Tumor Classification. IEEE Access, vol. 7, pp. 36266–36273, 2019. doi: 10.1109/ACCESS.2019.2904145
20. Haque R., Hassan M. M., Bairagi A. K., & S. Islam S. M. NeuroNet19: an explainable deep neural network model for the classification of brain tumors using magnetic resonance imaging data. *Scientific Reports*, 14(1), 2024. https://doi.org/10.1038/s41598-024-51867-1 PMID: 38233516
21. Hassan M. M., Haque R., Islam S. M. S., Meshref H., Alroobaea R., Masud M., et al. NeuroWave-Net: Enhancing epileptic seizure detection from EEG brain signals via advanced convolutional and long short-term memory networks. AIMS Bioengineering, 11(1), 85–109, 2024. https://doi.org/10.3934/bioeng.2024006
22. Ramamoorthy M., Qamar S., Manikandan R., Jhanjhi N. Z., Masud M., & AlZain M. A. Earlier Detection of Brain Tumor by Pre-Processing Based on Histogram Equalization with Neural Network. *Healthcare*, 10(7), 1218, 2022. https://doi.org/10.3390/healthcare10071218 PMID: 35885745
23. Joshi A. A., & Aziz R. M. Deep learning approach for brain tumor classification using metaheuristic optimization with gene expression data. *International Journal of Imaging Systems and Technology*, 2023. https://doi.org/10.1002/ima.23007
24. Yaqoob A., Verma N. K., & Aziz R. M. Optimizing Gene Selection and Cancer Classification with Hybrid Sine Cosine and Cuckoo Search Algorithm. *Preprint*, 2023. https://doi.org/10.21203/rs.3.rs-3357558/v1
25. Mahto R., Ahmed S. U., Rahman R. U., Aziz R. M., Roy P., Mallik S., et al. A novel and innovative cancer classification framework through a consecutive utilization of hybrid feature selection. *BMC Bioinformatics*, 24(1), 2023. https://doi.org/10.1186/s12859-023-05605-5 PMID: 38102551
26. Joshi A. A., & Aziz R. M. A two-phase cuckoo search based approach for gene selection and deep learning classification of cancer disease using gene expression data with a novel fitness function. *Multimedia Tools and Applications*, 2024. https://doi.org/10.1007/s11042-024-18327-4
27. CBIIT. Machine learning and computer vision offer a new way of looking at cancer. *CBIIT Blog*, 2021. Retrieved June 10, 2024, from https://datascience.cancer.gov/news-events/blog/machine-learning-and-computer-vision-offer-new-way-looking-cancer
28. Saxena A., Chouhan S. S., Aziz R. M., & Agarwal V. A comprehensive evaluation of Marine predator chaotic algorithm for feature selection of COVID-19. *Evolving Systems*, 2024. https://doi.org/10.1007/s12530-023-09557-2
29. Agrawal, R. An End-to-End Introduction to Generative Adversarial Networks (GANs). Available online: https://www.analyticsvidhya.com/blog/2021/10/an-end-to-end-introduction-to-generative-adversarial-networksgans/, June 2, 2023.
30. Sahoo Saswati, Mishra Sushruta, Panda Baidyanath, Bhoi Akash Kumar, Barsocchi Paolo. An Augmented Modulated Deep Learning Based Intelligent Predictive Model for Brain Tumor Detection Using GAN Ensemble. Sensors, vol. 23, no. 15, article number 6930, 2023. Available online: https://www.mdpi.com/1424-8220/23/15/6930. doi: 10.3390/s23156930 PMID: 37571713
31. Han C. and Rundo L. and Araki R. and Nagano Y. and Furukawa Y. and Mauri G. et al. Combining Noise-to-Image and Image-to-Image GANs: Brain MR Image Augmentation for Tumor Detection. IEEE Access, vol. 7, pp. 156966–156977, 2019. doi: 10.1109/ACCESS.2019.2947606
32. Ge, C. and Gu, I. Y. H. and Store Jakola, A. and Yang, J. Cross-Modality Augmentation of Brain Mr Images Using a Novel Pairwise Generative Adversarial Network for Enhanced Glioma Classification. 2019 IEEE International Conference on Image Processing (ICIP), September 2019.
33. Segmentation Labels and Radiomic Features for the Pre-operative Scans of the TCGA-LGG collection (BraTS-TCGA-LGG)—TCIA DOIs—Cancer Imaging Archive Wiki. Available online: https://wiki.cancerimagingarchive.net/pages/viewpage.action?pageId=24282668.

34. Kang J. and Ullah Z. and Gwak J. MRI-Based Brain Tumor Classification Using Ensemble of Deep Features and Machine Learning Classifiers. Sensors, vol. 21, no. 6, p. 2222, March 22, 2021. https://doi.org/10.3390/s21062222 PMID: 33810176
35. Brain MRI Images for Brain Tumor Detection. Available online: https://www.kaggle.com/datasets/navoneel/brain-mri-images-for-brain-tumor-detection, April 14, 2019.
36. Br35H :: Brain Tumor Detection 2020. Available online: https://www.kaggle.com/datasets/ahmed-hamada0/brain-tumor-detection, November 14, 2021.
37. Kaggle. Brain Tumor MRI Dataset. Published online, September 24, 2021. Available: https://www.kaggle.com/datasets/masoudnickparvar/brain-tumor-mri-dataset.
38. Kaggle. Brain Tumour Dataset. Published online, July 1, 2023. Available: https://www.kaggle.com/datasets/niranjananehru/brain-tumour-dataset.
39. Israel, Steven A., J.H. Goldstein, Jeffrey S. Klein, James Talamonti, Franklin Tanner, Shane Zabel, et al. Generative Adversarial Networks for Classification. In: 2017 IEEE Applied Imagery Pattern Recognition Workshop (AIPR), pages 1-4. 2017.
40. Gulrajani, Ishaan; Ahmed, Faruk; Arjovsky, Martin; Dumoulin, Vincent; Courville, Aaron. Improved Training of Wasserstein GANs. Year: 2017. ArXiv preprint arXiv:1704.00028.
41. Wang, Xintao, Ke Yu, Shixiang Wu, Jinjin Gu, Yihao Liu, Chao Dong, et al. ESRGAN: Enhanced super-resolution generative adversarial networks. In: The European Conference on Computer Vision Workshops (ECCVW). September 2018.
42. Wang, Xintao, Liangbin Xie, Chao Dong, and Ying Shan. Real-ESRGAN: Training Real-World Blind Super-Resolution with Pure Synthetic Data. In: International Conference on Computer Vision Workshops (ICCVW). 2021.
43. Simonyan, K., & Zisserman, A. Very Deep Convolutional Networks for Large-Scale Image Recognition. In *International Conference on Learning Representations*, 2015. https://doi.org/10.48550/arXiv.1409.1556
44. Bangar, S. VGG-Net Architecture Explained—Siddhesh Bangar—Medium. *Medium*, 2022, June 29. https://medium.com/@siddheshb008/vgg-net-architecture-explained-71179310050f
45. Huang G., Liu Z., Van Der Maaten L., & Weinberger K. Q. Densely Connected Convolutional Networks. In *CVPR*, vol. 1, no. 2, p. 3, 2017. https://doi.org/10.48550/arXiv.1608.06993
46. Szegedy, C., Ioffe, S., Vanhoucke, V., & Alemi, A. A. Inception-v4, inception-ResNet and the impact of residual connections on learning. In *Proceedings of the Thirty-First AAAI Conference on Artificial Intelligence*, 2017. AAAI Press, pages 4278–4284. https://doi.org/10.48550/arXiv.1602.07261
47. Schorr Christian, Goodarzi Payman, Chen Fei, and Dahmen Tim. Neuroscope: An Explainable AI Toolbox for Semantic Segmentation and Image Classification of Convolutional Neural Nets. In: Applied Sciences, volume 11, page 2199. March 2021. doi: 10.3390/app11052199
48. Shimanto SA, Hosain MK, Biswas SP, Islam MS. Brain tumor detection and classification by SVM algorithm and performance analysis through CNN approach. 2023.
49. Khan MSI, Rahman A, Debnath T, Karim MR, Nasir MK, Band SS, et al. Accurate brain tumor detection using deep convolutional neural network. Comput Struct Biotechnol J. 2022;20:4733–45. https://doi.org/10.1016/j.csbj.2022.08.039 PMID: 36147663
50. Díaz-Pernas FJ, Martínez-Zarzuela M, Antón-Rodríguez M, González-Ortega D. A deep learning approach for brain tumor classification and segmentation using a multiscale convolutional neural network. Healthcare (Basel). 2021;9(2):153. https://doi.org/10.3390/healthcare9020153 PMID: 33540873
51. Dipu NM, Shohan SA, Salam KMA. Deep learning based brain tumor detection and classification. 2021.
52. Saxena Priyansh, Maheshwari Akshat, Maheshwari Saumil. Predictive modeling of brain tumor: a deep learning approach. In: Predictive modeling and its applications in biomedical engineering, pp. 275–285, September 2020. ISBN: 978-981-15-6066-8.
53. Gudigar A, Raghavendra U, San TR, Ciaccio EJ, Acharya UR. Application of multiresolution analysis for automated detection of brain abnormality using MR images: A comparative study. Future Generation Computer Systems. 2019;90:359–67. https://doi.org/10.1016/j.future.2018.08.008
54. Hemanth DJ, Anitha J, Naaji A, Geman O, Popescu DE, Hoang Son L. A modified deep convolutional neural network for abnormal brain image classification. IEEE Access. 2019;7:4275–83. https://doi.org/10.1109/access.2018.2885639
